# Tumor characterization and stratification by integrated molecular profiles reveals essential pan-cancer features

**DOI:** 10.1186/s12864-015-1687-x

**Published:** 2015-07-07

**Authors:** Zhaoqi Liu, Shihua Zhang

**Affiliations:** National Center for Mathematics and Interdisciplinary Sciences, Academy of Mathematics and Systems Science, Chinese Academy of Sciences, Beijing, 100190 China

## Abstract

**Background:**

Identification of tumor heterogeneity and genomic similarities across different cancer types is essential to the design of effective stratified treatments and for the discovery of treatments that can be extended to different types of tumors. However, systematic investigations on comprehensive molecular profiles have not been fully explored to achieve this goal.

**Results:**

Here, we performed a network-based integrative pan-cancer genomic analysis on >3000 samples from 12 cancer types to uncover novel stratifications among tumors. Our study not only revealed recurrently reported cross-cancer similarities, but also identified novel ones. The macro-scale stratification demonstrates strong clinical relevance and reveals consistent risk tendency among cancer types. The micro-scale stratification shows essential pan-cancer heterogeneity with subgroup-specific gene network characteristics and biological functions.

**Conclusions:**

In summary, our comprehensive network-based pan-cancer stratification provides valuable information about inter- and intra- cancer stratification for patient clinical assessments and therapeutic strategies.

**Electronic supplementary material:**

The online version of this article (doi:10.1186/s12864-015-1687-x) contains supplementary material, which is available to authorized users.

## Background

Cancer largely results from various molecular aberrations comprising somatic mutational events such as single nucleotide mutations, copy number changes and DNA methylations [1–3]. In addition, cancer is viewed as a wildly heterogeneous disease, consisting of different subtypes with diverse molecular implementations of oncogenesis and therapeutic responses. Many organ-specific cancers have established definitions of molecular subtypes on the basis of genomic, transcriptomic, and epigenomic characterizations [1–3], indicating diverse molecular oncogenic processes and clinical outcomes. The molecular-defined intrinsic breast cancer subtypes (luminal A, luminal B, HER2-enriched, basal-like, and normal-like) are typical examples, since they have been reported to be associated with distinct phenotype outcomes and have different chemotherapy responses and respective stratified therapy [4–8]. Similarly, endometrial cancers have also been classified into four categories (POLE ultramutated, microsatellite instability hypermutated, copy-number low, and serous-like) through a comprehensive, multiplatform analysis [9], and glioblastoma multiformae was stratified into four distinct molecular subtypes (proneural, neural, classical, and mesenchymal) based on the CpG island methylation phenotype [1]. Different tumor subtypes of the same organ reflect diverse molecular oncogenic processes and various clinical outcomes, which imply that they should be treated as different cancers for treatment design in some sense [10].

Key genomic similarities shared by subgroups of patients across cancer types would present an opportunity to design tumor treatment strategies among tumors regardless of tissue or organ of origin and enable the extension of effective treatments from one cancer type to another [11]. For example, the molecular commonalities between basal-like breast tumors with high-grade serous ovarian tumors indicate a related etiology and similar therapeutic opportunities [12]. However, the current tumor heterogeneity is mostly defined for tumors of the same organ without considering the potential cross-cancer benefits. Thus, deciphering tumor heterogeneity for all cancers based on their genomic characteristics is an urgent issue.

In the past, insufficiency of high quality genomic datasets of a large number of patients across different tumor types has impeded such investigations. With great advancement in high-throughput sequencing technologies and comprehensive efforts of systematic cancer genomics projects (e.g., the Cancer Genome Atlas pan-cancer project [11]), studies on molecular aberrations of cancer patients have increased unprecedentedly in scale and accessibility, enabling large-scale integrative cross-cancer analysis [13]. Very recently, Hoadley *et al*. conducted a comprehensive integrative analysis using data from six independent omics platforms on 3,527 specimens from 12 cancer types and reported a unified classification into 11 major subtypes (originally, there were 13 classes and 2 classes only had 3 samples and 6 samples respectively) [14].

Cancer has long been considered as a disease of combinations of functionally related alterations at the network level. In recent years, the molecular network as a simple but efficient presentation of complex interactions and regulatory relationships between molecules has been adopted comprehensively for understanding system-level properties of complex disease. However, Hoadley *et al*. only adopted very limited information on pathways and failed to employ a large-scale molecular interaction network [14]. In contrast, we believe that aggregating genomic characterizations of patients using gene networks would contribute to identifying subgroups of patients with similar molecular-network patterns affected by diverse genetic alterations.

In this study, we adopted a network-based stratification (NBS) approach [15] to integrate key genetic and epigenetic features of 3299 tumor samples from 12 cancer types [16] to uncover novel pan-cancer heterogeneity. We found that our pan-cancer stratification is predictive of clinical outcomes, and different cancer patients falling into the same subgroup show consistent survival tendency or grade/stage severity. We identified subgroup-specific genomic alterations and networks that are responsible for distinguishing each subgroup. These subgroup networks demonstrate specific genomic characteristics and biological functions. In summary, our cross-cancer stratifications not only revealed most recurrently reported cross-cancer similarities, but also novel patient groupings, implying valuable messages for patient clinical assessments and therapeutic strategies.

## Results

### Overview of the pan-cancer stratification analysis

**Fig. 1:**
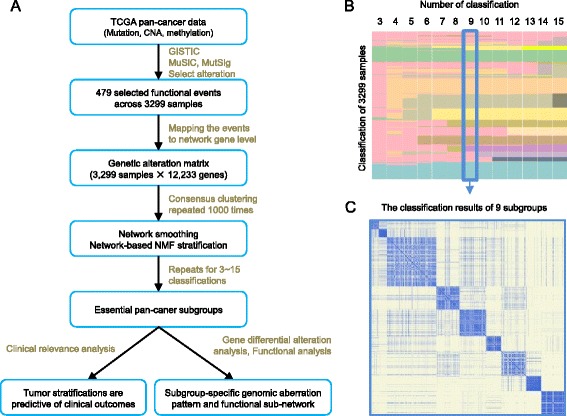
Overview of the pan-cancer stratification analysis. **a** Flowchart of the main computational procedure. **b** The landscape of pan-cancer subgroups with *k* = 3 ~ 15 classes obtained from the consensus NBS clustering. Each row denotes a sample, and each column presents a classification. Different colors in each column denote different subgroups. **c** The heat map of the co-clustering matrix of the 9 subgroups obtained from the consensus NBS clustering

We integrated and mapped the genomic aberrations of tumors of 12 cancer types to a large-scale molecular interaction network, and adopted the NBS procedure to reveal pan-cancer subgroups with similar molecular features (see Methods, Fig. 1 and in Additional file 1: Figure S1). The 12 cancer types include bladder urothelial carcinoma (BLCA), breast invasive carcinoma (BRCA), colon and rectum adenocarcinoma (COAD, READ), glioblastoma multiformae (GBM), head and neck squamous cell carcinoma (HNSC), kidney renal clear-cell carcinoma (KIRC), acute myeloid leukemia (LAML), lung adenocarcinoma (LUAD), lung squamous cell carcinoma (LUSC), ovarian serous cystadenocarcinoma (OV), and uterine corpus endometrioid carcinoma (UCEC) (in Additional file 1: Table S1).

We can observe clear consistency between every successive two classifications (e.g., *k* = 6 versus *k* = 7) of the samples (Fig. 1b). In particular, two patient subgroups were consistently identified across all 3 ~ 15 classes (samples denoted by light blue and green in Fig. 1b). One subgroup was dominated by KIRC tumors. KIRC has been reported to have a high frequency of Von Hippel-Lindau (VHL) mutation and show distinct exclusivity from other 11 cancer types [17]. The other subgroup consists of subsets of GBM, BLCA, LUSC, and HNSC tumors. The similarity of these tumors has been implicated in the mutation or amplification of *ERBB2-HER2* [11]. The remaining patients are progressively subdivided into new subgroups as the number of classes gets larger. We will further explore those representative subgroups in terms of macro-scale (with *k* = 3) and micro-scale (with *k* = 9 and Fig. 1c) classes in the following subsections.

### Macro-scale pan-cancer subgroups reveal clinical relevance

**Fig. 2:**
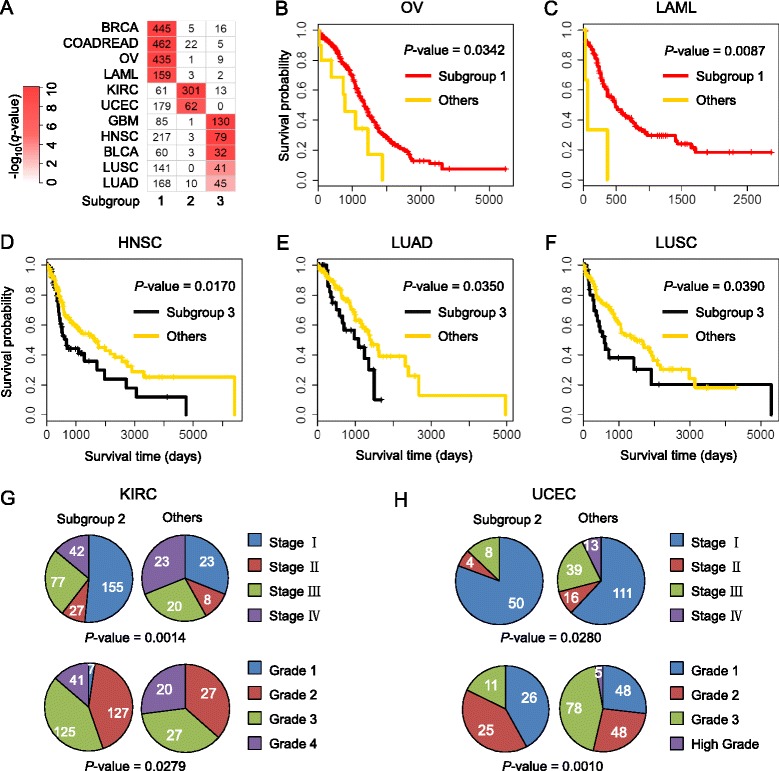
Macro-scale pan-cancer subgroups reveal clinical relevance. **a** Patient distribution of 12 cancer types (COAD and READ are treated as one type) in pan-cancer subgroups with *k* = 3. For a patient set of each cancer type in a subgroup, the significance of enrichment was evaluated using a chi-squared test (see Methods). *P*-values lower than 1 × 10^−10^ were set as 1 × 10^−10^ for convenient visualization. The number denotes the size of the corresponding patient cohort of each cancer type in this subgroup. **b-f** Kaplan-Meier survival curves of patients in significantly enriched subgroups and the remaining ones for cancer types OV **b**, LAML **c**, HNSC (**d**), LUAD (**e**), and LUSC **f** were plotted, respectively. *P*-values were derived from the log-rank test. **g-h** Pie plots demonstrating the distributions of tumor stage and grade on patients in significantly enriched subgroups and others for cancer types KIRC (**g**) and UCEC **h**. *P*-values denoting the significant level of the difference between the distributions of two groups were calculated by Fisher’s exact test

We found that the unsupervised macro-scale pan-cancer subgroups (with *k* = 3) reveal distinct clinical relevance across diverse cancer types (Fig. 2). We first observed that each cancer type was significantly clustered into one of the three pan-cancer subgroups (Fig. 2a). We further found that the significantly enriched patients of five cancer types demonstrated significantly different survival rates compared to the remaining patients of the same cancer types, respectively (Fig. 2b-f). In particular, the patients for OV and LAML in subgroup 1 are associated with long survival time and those for HNSC, LUAD, and LUSC in subgroup 3 are correlated with bad survival outcomes. More intriguingly, we found that patients in subgroup 3 tend to have relatively poorer survival for almost all cancer types, and COADREAD and OV subgroup 3 patients also show statistically significant shorter survival time (log-rank *p*-value <0.05) (in Additional file 1: Figure S2). Similarly, subgroup 1 patients were associated with better survival outcomes for almost all cancer types and HNSC, LUAD, and LUSC show statistical significance (in Additional file 1: Figure S3).

Lastly, we found that a large fraction of KIRC tumors and a subset of UCEC tumors were significantly enriched in subgroup 2 (Fig. 2a). Those KIRC tumors and UCEC tumors in subgroup 2 tend to be patients at early tumor stage and low grade (Fig. 2g, h). More than half of the KIRC tumors in subgroup 2 are at Stage I, and no UCEC tumor in subgroup 2 is at Stage IV and high grade. All these observations demonstrate that our pan-cancer macro-scale stratification reveals strong clinical relevance and shows consistent clinical tendency in some cancer types, implying distinct pan-cancer heterogeneity as well as oncogenic mechanisms.

Hoadley *et al*. [14] reported patient overall survival of their 11 identified subtypes, which is very similar to the overall survival observed in the original cancer types, indicating limited contribution to the prognosis evaluation and stratified therapy of patients. However, we note that the comparison of patient survival among cancer tissue types is limited to some extent. For example, GBM or LAML patients are often associated with poor prognosis compared to relatively moderate BRCA or UCEC patients. Seen from this angle, our pan-cancer macro-scale stratification divides almost all cancers into subgroups with consistent good or poor survival rates, revealing underlying pan-cancer similarities among cancer types and providing valuable information for patient clinical assessments and stratified therapeutic strategies.

### Micro-scale pan-cancer subgroups reveal abundant cross-cancer similarities

**Fig. 3:**
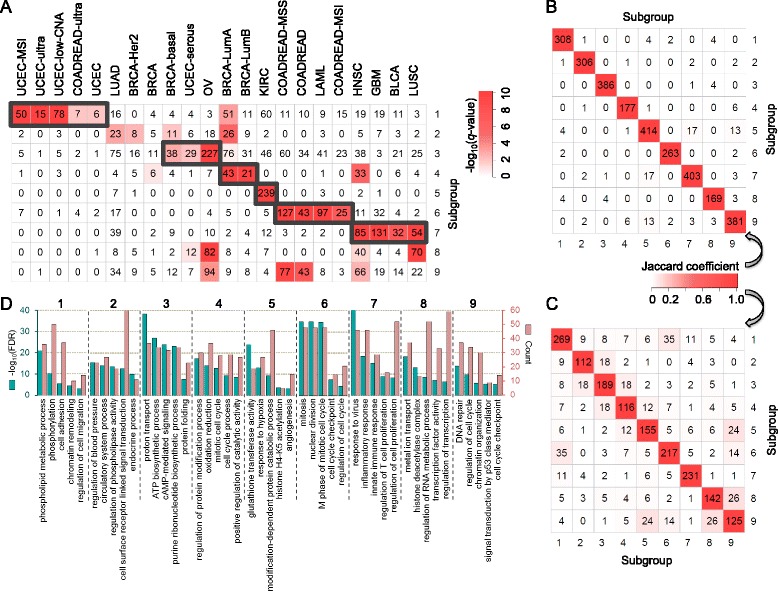
Micro-scale pan-cancer subgroups demonstrate distinct subgroup-specific patterns. **a** Patient distribution of 12 cancer types (COAD and READ are treated as one type) in pan-cancer subgroups with *k* = 9. Subtypes of three cancers were used. For example, BRCA-Her2 stands for the Her2+ breast cancer subtype, while BRCA stands for the breast cancer samples with no subtype information. For a patient set of each cancer type or subtype in the subgroups, the significance of enrichment was evaluated using a Chi-squared test (see Methods). *P*-values lower than 1 × 10^−10^ were set as 1 × 10^−10^ for convenient visualization. The number denotes the size of the corresponding patient cohort of each cancer type or subtype in this subgroup. **b** The number of overlaps between each pair of the 9 sets of significantly differentially influenced genes in each subgroup. **c** The number of overlaps of the biological functional terms derived from the corresponding significantly differentially influenced genes sets of each subgroup using DAVID. Genes and functional terms were selected with false discovery rate (FDR) *q*-value smaller than 0.05. **d** Selected GO terms (biological processes) from the functional analysis using the 9 sets of significantly differentially influenced genes for each subgroup. Bars represent the significance with -log_10_(FDR) (green) and the number of enriched genes (red) of the corresponding GO term

Further, we found that the micro-scale pan-cancer subgroups (e.g., with *k* = 9) reveal heterogeneous aberration patterns across diverse cancer types. (For convenience, we named all subgroups as PC9 subgroup-X, X = 1… 9, or subgroup-X for short; Fig. 3) We observed that most of the 12 cancer types and their subtypes were significantly clustered into at least one of the 9 pan-cancer subgroups (Fig. 3a).

We first found that 94.4 % of the tumors in subgroup-5 were KIRC types, making this subgroup highly exclusive to a single cancer type, and more than half of tumors (56.8 %) in subgroup-4 were BRCA types. In contrast to these two subgroups dominated by individual cancer type, other subgroups consist of multiple cancer types. For example, subgroup-7 is significantly enriched with a large fraction of GBM (60.6 %), HNSC, LUSC, and BLCA tumors. In subgroup-6, 59.1 % of LAML tumors and three molecule-defined COADREAD subtypes were clustered together, indicating potential commonalities between solid and liquid tumors (Fig. 3a).

We next explored the network modules consisting of significant differentially influenced genes for each subgroup (see Methods). We can see that the overlap of these gene sets is very limited, indicating that these gene sets are highly specific to a subgroup (Fig. 3b). Moreover, the biological functional annotations of these 9 gene sets are also very specific to individual subgroups (Fig. 3c, d).

### Micro-scale pan-cancer subgroups demonstrate distinct subgroup-specific patterns

**Fig. 4:**
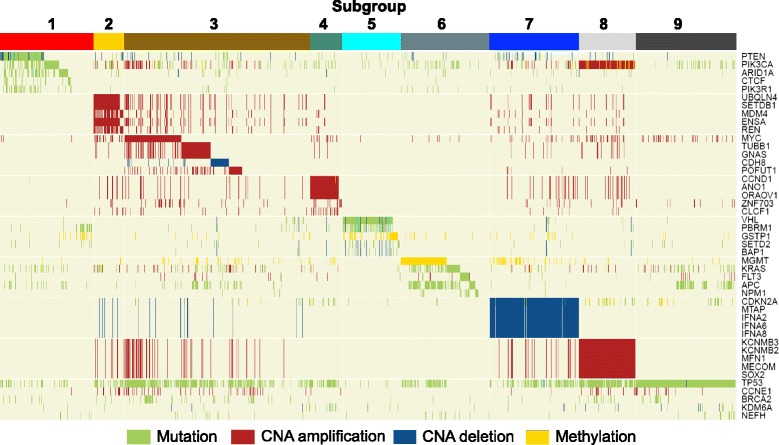
Landscape of genomic alteration patterns of pan-cancer subgroups. Landscape of genomic alteration patterns of each pan-cancer subgroup with *k* = 9. Each row denotes a gene and each column indicates a sample. Different colors on the top band denote the 9 pan-cancer subgroups. Different colors in the map indicate different kinds of genomic aberrations. The more detailed genomic landscapes for each subgroup are presented in Figures S4-S12 in Additional file 1

**Fig. 5:**
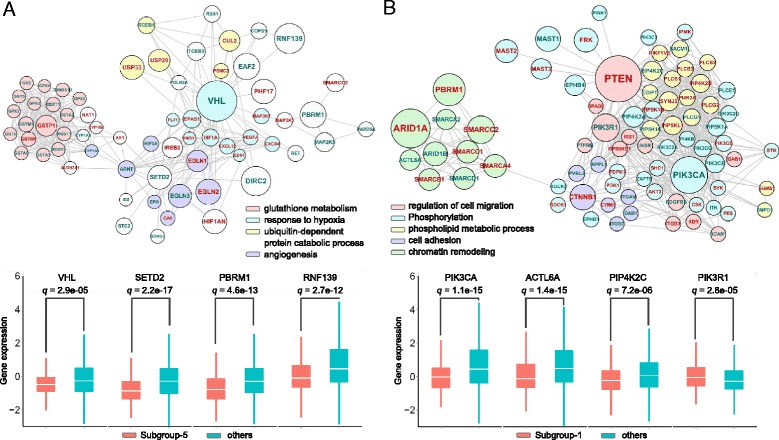
Subgroup-specific molecular network characteristics for subgroup-5 and subgroup-1. Network modules consisting of significant differential altered genes of subgroup-5 (**a**) and subgroup-1 (**b**). The node size denotes the network propagation score from the NBS, showing the effect of genetic alterations in the biological network. The node color corresponds to different biological functional terms. The node labels of genes with significant mRNA expression changes (*q*-value < 0.05) in each subgroup compared to other patients are marked with green. Below the network module, four significantly differential expressed genes in this subgroup compared to the remaining ones are demonstrated for each subgroup. The significance of differential expressions of the altered genes was calculated using the Wilcoxon rank-sum test (with corrected *q*-value < 0.05). Similar settings were used for Fig. 6

More importantly, genes from each gene set with high aberration frequencies among corresponding subgroups indeed show significantly distinct patterns among the 9 subgroups (Fig. 4). These observations imply that diverse carcinogenic implementations and functional genetic alteration events exist in different pan-cancer subgroups, depicting essential tumor heterogeneity. More specifically, the KIRC-specific subgroup-5 possesses exclusive somatic mutation of the tumor suppressor gene *VHL* with a mutation rate of 81.8 % in subgroup-5 (Fig. 4 and Fig. 5a) [17]. The relationship between mutations of *VHL* and KIRC has been established for decades and the association between *VHL* and tumor stage, tumor-cell proliferation, and patient prognosis has also been well studied [18, 19]. Besides *VHL*, other genetic alterations in subgroup-5 involve the mutation of the chromatin remodeling gene *PBRM1*, the mutation of the histone methyltransferase gene *SETD2*, which has been identified as a tumor suppressor in KIRC [20] and high methylation rate of *GSTP1* (Fig. 4 and in Additional file 1: Figure S8). Moreover, *VHL*, *SETD2*, *PBRM1*, and others display significant low expression in this subgroup compared to the remaining ones (Fig. 5a). These genomic alterations in this subgroup are exclusive to KIRC, marking it highly exclusive from other cancer types.

Multiple cancer types or subtypes including COADREAD-ultra and UCEC as well as BRCA-luminal A tumors are significantly enriched in pan-cancer subgroup-1 (Fig. 3a). This subgroup was marked by mutations of multiple genes that exhibit a mutually exclusive pattern in this cohort (Fig. 4 and in Additional file 1: Figure S4). Both *PTEN* and *PIK3CA* alterations were reported to have strong relationships with UCEC and COADREAD, and the loss of *PTEN* expression is also observed to be associated with PIK3CA mutations in metastatic colorectal cancer [21–25]. Altered *PTEN* expression was viewed as a diagnostic marker for early detection of UCEC [21], and is associated with favorable clinical and pathologic characteristics [22]. In addition, *PIK3CA* mutations were reported to be present in approximately 25 % of breast cancers, particularly the estrogen receptor–positive subtypes, while they are absent in the basal-type breast cancer [26]. This is consistent with the fact that luminal A breast tumors are significantly enriched in this subgroup. The mutation of *PTEN* and *PIK3CA* together with other alterations of genes affects a common biological network, which reflects the major similarities among subgroup-1 tumors (Fig. 5b in Additional file 1). Moreover, high methylation frequency of *MLH1* was observed exclusively in the UCEC-MSI cohort of subgroup-1 (in Additional file 1: Figure S4), confirming that *MLH1* promoter methylation is the primary cause of microsatellite instability in sporadic endometrial cancers [27]. Finally, many subgroup-1-specific altered genes including *PIK3CA* show significant differential expression in subgroup-1 compared to all other patients (Fig. 5b), indicating the potential associations with downstream expression changes.

**Fig. 6:**
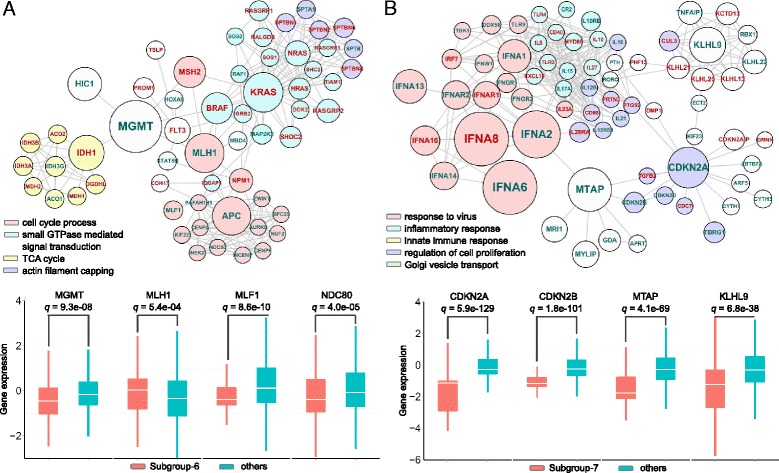
Subgroup-specific molecular network characteristics for subgroup-6 and subgroup-7. Network modules consisting of significant differential altered genes of subgroup-6 (**a**) and subgroup-7 (**b**)

Subgroup-6 was mainly characterized by frequent promoter hypermethylation of *MGMT* and mutations of *APC*, *KRAS*, *FLT3*, and *NPM1* (Fig. 4, Fig. 6a and in Additional file 1: Figure S9). Patients in this subgroup contain 40.6 % COADREAD and 59.1 % LAML as well as sporadic samples from other types (Fig. 3a). About one-fifth of LAML samples in subgroup-6 were described with *MGMT* methylation. Increased *MGMT* activity is associated with resistance to cancer therapy using an alkylating agent, temozolomide, which has been shown to inhibit cell growth in leukemia cell lines [28]. Thus, hypermethylation of *MGMT*, inhibiting the expression of this gene, is of clinical interest for LAML. We indeed observed that it showed significant lower expression in this subgroup than in others (Fig. 6a). Moreover, the methylation of *MGMT* was also reported as a valuable molecular marker for the early detection of colorectal cancer [29]. Therefore, the alteration of *MGMT* would provide potential implications for targeted and shared therapy across these two malignancies. Besides *MGMT*, these two solid and liquid tumors also share other mutated genes including *KRAS*, *IDH1*, and *NRAS*. In contrast, we also found that a few genes are tumor-specific for patients in subgroup-6. For example, mutations of tumor suppressor gene *APC* were only presented in COADREAD, while mutations of *FLT3* and *NPM1* are exclusive to LAML.

Subgroup-7 was characterized by the copy number deletion on chromosome 9p21 (98.4 % CNA deletion; Fig. 4 and in Additional file 1: Figure S10). Genes located in this region include *CDKN2A*, *CDKN2B*, *KLHL9*, and *MTAP* as well as the *IFNA* gene family. More than half of GBM (60.6 %) were clustered in subgroup-7 with other significant enriched cancer types of HNSC, LUSC, and BLCA (Fig. 3a and Fig. 6b in Additional file 1). This subgroup demonstrates a typical cross-cancer similarity phenomenon that subsets of samples from different tumor types are characterized by the same genomic alterations on chromosome 9. The associations of the deletion of tumor suppressor genes *CDKN2A*, *CDKN2B,* and *MTAP* with the four significant enriched cancer types in this subgroup have been widely investigated and reported [30–34]. *IFNA1*, *2*, *6*, *8*, *9*, and *13* are members of the alpha-interferon genes cluster on chromosome 9. Interferons are encoded by *IFNA* genes in response to the presence of pathogens such as viruses, bacteria, parasites, or tumor cells. They activate immune cells, trigger the protective defenses of the immune system, and eradicate pathogens or tumors. As is known, viruses cause 10–15 % of all human cancers, and inflammation promotes oncogenesis in the evolution of cellular transformation [35, 36]. It was reported that human papilloma virus (HPV) types 16 and 18 were detected in HNSC and played an important role in carcinogenesis of this cancer [37]. Similar discoveries show that HPV is the second most important cause of lung cancer after cigarette smoking [38]. Shokeir *et al*. [39] showed that the carcinogenesis of bladder cancer is likely related to bacterial and viral infections. In addition, another study also suggested that HPV infection status could be considered as an independent prognostic factor for GBM and recognized as a causative agent in gliomagenesis [40]. The lack of expression due to the deletion of *IFNAs* may be responsible for the HPV infection in carcinogenesis of these cancers; however, their relationships need to be further investigated. Subgroup-7 has shown distinct gene expression differences such as that of *CDKN2A*, *CDKN2B*, *MATP*, *KLHL9*, *IFNA2*, and *IFNA6* with extremely low q-values, which could be explained by the ~100 % copy number deletion on chromosome 9 in subgroup-7 (Fig. 6b).

Subgroup-2 mainly consists of LUAD and BRCA tumors, which were characterized by the amplifications on chromosome 1 involving *UBQLN4*, *SETDB1*, *MDM4*, *ENSA*, and so forth (in Additional file 1: Figure S5). The largest patient group, subgroup-3 enriched with BRCA-basal, UCEC-serous, and OV tumors, was characterized by multiple recurrent chromosomal gains and losses (in Additional file 1: Figure S6A). The amplification of oncogene *MYC* occurs in 30.8 % of samples in subgroup-3. BRCA-basal, UCEC-serous, and OV patients in this cohort are associated with a high mutation rate of *TP53* (88.4 %) (in Additional file 1: Figure S6B), which was consistent with previous observations [11, 14]. Amplification of 11q13 involving *CCND1*, *ORAOV1*, and *ANO1* was dominated in subgroup-4, mainly consisting of luminal BRCA and HNSC (in Additional file 1: Figure S7). These estrogen-receptor positive luminal tumors are significantly enriched in this subgroup, while basal-like breast cancers are not. Amplification and overexpression of *CCND1* would alter cell cycle progression and contribute to tumorigenesis. Previous studies have shown that luminal cancers harbor recurrent amplifications and overexpression of *CCND1*, whereas basal-like tumors harbor recurrent deletions and down-regulation of it [41, 42]. Subgroup-8, mainly consisting of LUSC, HNSC, and OV tumors, was characterized by 100 % copy number gain on chromosome 3q26 involving genes *PIK3CA*, *KCNMB3*, *KCNMB2*, *MFN1*, *GNB4*, *MECOM*, *ZMAT3*, *SOX2*, and *KCNJ13* (in Additional file 1: Figure S11). Subgroup-9, mainly consisting of HNSC, OV, and COADREAD, was characterized by a distinct *TP53* mutation rate (98.6 %, in Additional file 1: Figure S12).

## Discussion

In this paper, we adopted a network framework to integrate the alteration profile of 12 cancer types to reveal essential pan-cancer heterogeneity among diverse cancers. Without considering the primary tumor organ information, all tumors were clustered into pan-cancer subgroups, which allowed us to discover important cross-cancer commonalities. In a recent study, Ciriello *et al*. [16] revealed two major classes, the M class (dominated by mutation) and the C class (dominated by CNAs), and further derived a hierarchical classification of patients based on the binary event data by repeating the algorithm on each newly identified class. However, this process affects the identification of tumor heterogeneity and ignores the cross-cancer similarities embodied in pathways and networks. Our network-based stratification can conquer these limitations of the sparsity of the discrete binary data and the lack of information on neighboring genes.

**Fig. 7:**
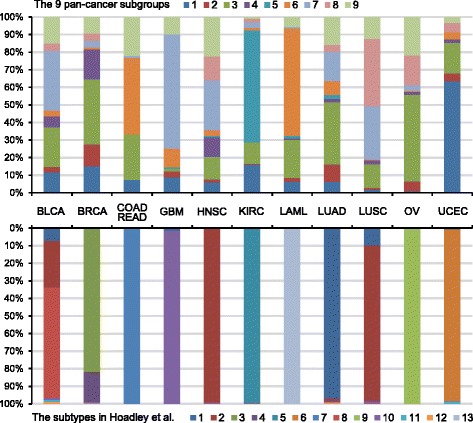
The distributions of the 12 cancer types (COAD and READ were treated as one type) under our pan-cancer classification and that of Hoadley *et al*. Above: our pan-cancer classification (PC9). Below: the pan-cancer classification by Hoadley *et al*. 2631 samples were involved in both classifications for this comparative analysis

More recently, Hoadley *et al*. identified 11 major pan-cancer classes by integrating the data of six platforms. However, these classes are highly correlated to the cancer tissue of origin, revealing limited features shared by diverse cancers. In contrast, our stratifications uncover distinct cross-cancer similarities and significant clinical relevance (Figs 2 and 3, Fig 7 and in Additional file 1: Figure S13). Hoadley *et al*. clustered the combined hard membership matrices of unsupervised clustering results from all data platforms to get the final classification, which ignores the distinct diversity of each data and oversimplifies the underlying clustering features. Moreover, Hoadley *et al*. did not make full use of the effect of mutated genes on their neighboring genes through large-scale biological networks.

Specifically, among the 11 classes identified by Hoadley *et al*., five show near one-to-one relationships with tissue of origin, while only one subgroup was found in our PC9 subgroups (KIRC specific subgroup-5; in Additional file 1: Figure S13). This repeated finding further confirms the highly exclusive molecular characteristics of KIRC compared to others. We also clustered BRCA luminal tumors and basal-like tumors into two separate classes (subgroup-3 and subgroup-4) as done by Hoadley *et al*., emphasising the intrinsic divergence of this tumor (in Additional file 1: Figure S13). The most important cross-cancer class in Hoadley *et al*. [14] is the squamous-like subtype, which consists of LUSC, BLCA, and some BLCA. Similar observations in our work can be found in subgroup-7 with additional enriched GBM samples (in Additional file 1: Figure S13). Both studies reported the loss of CDKN2A in this patient cohort; however, our subgroup-7 was characterized by the copy number deletion on chromosome 9p21 with nearly 100 % frequency. We also found that the loss of *IFNA* family genes in this group may be related to the virus infection in carcinogenesis of these tumors. Our results revealed the known cross-cancer similarities between basal-like and serous OV, however, which was failed to be clustered together in Hoadley *et al*. [12, 14] (in Additional file 1: Figure S13). In addition, our study reveals more cross-cancer similarities that were not reported in Hoadley *et al*. such as the hypermethylation of *MGMT* and other genetic characteristics shared by subsets of LAML and UCEC in subgroup-6 and the 100 % copy number gain on chromosome 3q26 in fractional OV, LUSC, and HNSC in subgroup-8 (in Additional file 1: Figure S13).

Finally, in order to evaluate the robustness of our classification to obtain the 9 pan-cancer subgroups, we performed random subsamplings of the samples and reclassified the reduced dataset into 9 classes with the same calculation procedure. The results demonstrate that our pan-cancer stratification is a robust grouping system that can uncover very consistent patient assignments (in Additional file 1: Figure S14 ).

## Conclusions

In summary, our comprehensive network-based stratification of 12 cancer types reveals essential pan-cancer heterogeneity among diverse cancers without considering the primary tumor organ information. The uncovered similarities among cancers of different organs suggest important cross-cancer commonalities. These commonalities not only cover most of the recurrently reported cross-cancer similarities, but also identify several novel potential ones. The macro-scale pan-cancer subgroups demonstrate strong clinical relevance and reveal consistent clinical risk tendency among cancer types. The micro-scale stratification shows essential pan-cancer heterogeneity with subgroup-specific genomic network characteristics and molecular implementations of oncogenesis. We believe that the pan-cancer subgroups defined here are promising stratifications of tumors for deciphering the underlying mechanisms of cancer deeply. With the rapid accumulation of cancer genomics data, this pan-cancer subgrouping procedure can be adopted for a more comprehensive understanding of the pan-cancer heterogeneity. Moreover, it is known that mutations in the same gene can lead to different consequences depending on which domain interface is altered [43–45]. How to integrate such information into the pan-caner stratifications is of great interest and worth exploring in further study.

## Methods

### Functional genetic alterations data

We obtained the 479 selected functional events (SFEs) of three data types (copy number alterations, somatic mutations, and DNA hyper-methylations) that were filtered by statistical and functional significant analysis from thousands of genomic and epigenetic changes [16]. The SFEs binary data were downloaded from http://cbio.mskcc.org/cancergenomics/pancan_tcga/. These data contain 479 functional genetic alterations, including 116 copy number gains, 151 copy number losses, 199 recurrently mutated genes, and 13 epigenetically silenced genes recorded across 3299 tumor samples from 12 cancer types (Additional file 1: Table S1). Three cancer types (breast, colorectal, and endometrioid tumors) were provided with molecular subtype information. The profile is represented by binary (1, 0) values, in which a “1” indicates that a certain genetic alteration has occurred in this tumor.

### Data preprocessing

We first transformed the 479 functional genetic changes to genes. The genes located in the same region of recurrent copy number gain and loss were treated equally as altered events. Secondly, multiple alterations on the same gene (e.g., a gene was observed to harbor both copy number gain and mutation) were merged. This resulted in a binary matrix of 3299 samples with 1750 genes, where a “1” means the gene has been altered by some kind of genomic or epigenetic change. Finally, genes were projected onto a biological network STRING v.9 [46] and gene symbols were mapped to Ensembl IDs for downstream analysis (in Additional file 2).

### Identifying essential cancer subgroups using NBS

We adopted the NBS procedure [15] to integrate a genome-scale alteration profile with a gene interaction network (STRING v.9) to produce robust classifications of patients (in Additional file 2). Briefly, the NBS applies a network propagation method to spread the influence of each mutation over its network neighborhood and produce a network-smoothed profile to reflect the effect of each genetic alteration on network module or pathway levels with a continuous value. Next, the network-smoothed patient matrix is clustered into a predefined number of subgroups via a network-regularized non-negative matrix factorization approach. Finally, in order to ensure robust cluster assignments, consensus clustering was performed. We employed the MATLAB package “nbs_release_v0.2” (http://chianti.ucsd.edu/~mhofree/wordpress/?page_id=26) to implement NBS to stratify samples into *k* (*k* = 3 ~ 15) clusters (in Additional file 2: Table S2). All other parameters were set as defaults. We adopted the Pearson’s chi-squared test to determine the enrichment significance of a certain tumor type or subtype in a cluster. All *P* values were corrected for the FDR *q* value.

### Clinical outcome association analysis

We test to see if the identified subgroups are associated with clinical features of a specific cancer type including patient survival, tumor grade, and stage. The clinical data of 12 cancer types were downloaded from the TCGA_Pancancer page on Synapse (https://www.synapse.org/#!Synapse:syn300013/). Patient survival time was extracted from the tab-separated *.patient* files and detailed AJCC TNM staging information was merged (e.g., Stage IIA/IIB/IIC was merged as Stage II). Patients with missing clinical variables were excluded from the correlation analysis for that feature. For each cancer type, the survival information of samples located in different cohorts (e.g., BRCAs in its enriched subgroup versus all other BRCAs) was compared using Kaplan-Meier survival curves with log-rank test. The association of tumor grade/stage annotation with identified tumor subgroups was evaluated by Fisher's exact test. We conducted these analyses for each cancer type individually. Survival analysis was conducted using the R package “survival” and “survcomp”.

### Identifying differentially altered sub-networks for each pan-cancer subgroup

For patients in each subgroup, we identified significantly altered genes against the remaining samples based on the network-smoothed alteration data by SAM (SAM—significance analysis of microarrays—was originally designed for identifying differentially expressed genes) [47]. The *q*-value was calculated using the SAM permutation scheme with 1,000 permutations. The top significantly altered genes (SAM score >15 and FDR *q*-value <0.05) in each subgroup were selected as “significant differentially influenced genes”, and were mapped to the STRING v.9 network for visualization using the Cytoscape software. The biological functional analysis of the “significant differentially influenced genes” in each subgroup was performed using DAVID (http://david.abcc.ncifcrf.gov/) and GeneMANIA (http://www.genemania.org/). Annotation categories were pre-selected as defaults in DAVID and only terms with *q*-values lower than 0.05 were selected.

### Identifying genes with subgroup-specific mRNA expression changes

We adopted the normalized RNA Seq V2 RSEM data of the 3299 TCGA samples for identifying genes with significant subgroup-specific expression changes. The dataset was downloaded from the cBioPortal for Cancer Genomics (http://www.cbioportal.org/public-portal/index.do) using the R package “cgdsr.” For GBM and OV, we used Agilent microarray data instead since it covers more patients presented in the SFEs binary dataset. For each PC9 subgroup, gene expressions were compared using the Wilcoxon rank-sum test on patients in this subgroup and those in the remaining subgroups. We conducted this analysis for all differentially altered genes of each subgroup. *P* values were corrected to get the *q-*values using Benjamini and Hochberg correction [48].

## Additional files

Additional file 1:
**This file contains supplementary text, legends of supplementary figures and tables, and supplementary figures and tables.**
Additional file 2: Table S2. The subgroup assignments of TCGA patients across *k* = 3 ~ 15 classifications. 104 patients with no alterations observed in their profiles (selected functional events, SFEs data) were excluded from the list of 3299 patients. **data.mat.** The well pre-processed SFEs data used in this work. **code.m.** Code for calculating the pan-cancer stratifications using the NBS method in this work.
