# Supplementary material for: Tumor characterization and stratification by integrated molecular profiles reveals essential pan-cancer features
Source: BMC Genomics. 2015 Jul 7;16(1):503. doi: 10.1186/s12864-015-1687-x (PMC4491878; doi:10.1186/s12864-015-1687-x)
Supplement: Additional file 1: — This file contains supplementary text, legends of supplementary figures and tables, and supplementary figures and tables. [file 12864_2015_1687_MOESM1_ESM.pdf]

Supplementary Materials for

Comprehensive pan-cancer stratification analysis of key genetic and epigenetic features reveals essential tumor heterogeneity

Zhaoqi Liu, Shihua Zhang*

National Center for Mathematics and Interdisciplinary Sciences, Academy of Mathematics and Systems Science, Chinese Academy of Sciences, Beijing 100190, China
*Corresponding author. Email: zsh@amss.ac.cn

Table S1. Summary of the TCGA datasets adopted in this work.

Cancer type	Abbreviation	The SFEs binary calls data	Clinical feature data	mRNA expression data
Bladder urothelial carcinoma	BLCA	97	85	95
Breast invasive carcinoma	BRCA	488	458	465
Colon and rectum adenocarcinoma	COADREAD	491	369	251
Glioblastoma multiformae	GBM	218	213	200
Head and neck squamous cell carcinoma	HNSC	302	299	297
Kidney renal clear-cell carcinoma	KIRC	420	418	372
Acute myeloid leukemia	LAML	184	162	144
Lung adenocarcinoma	LUAD	229	200	223
Lung squamous cell carcinoma	LUSC	182	171	181
Ovarian serous cystadenocarcinoma	OV	446	443	438
Uterine corpus endometrioid carcinoma	UCEC	242	242	233

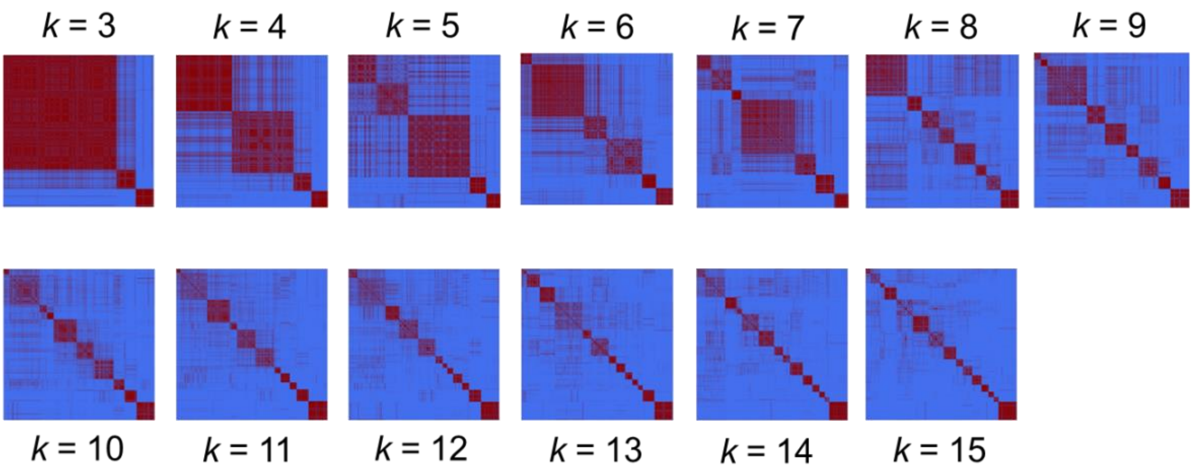

Figure S1. Heat maps of the co-clustering matrix of k = 3~15 classifications obtained from the consensus NBS clustering.

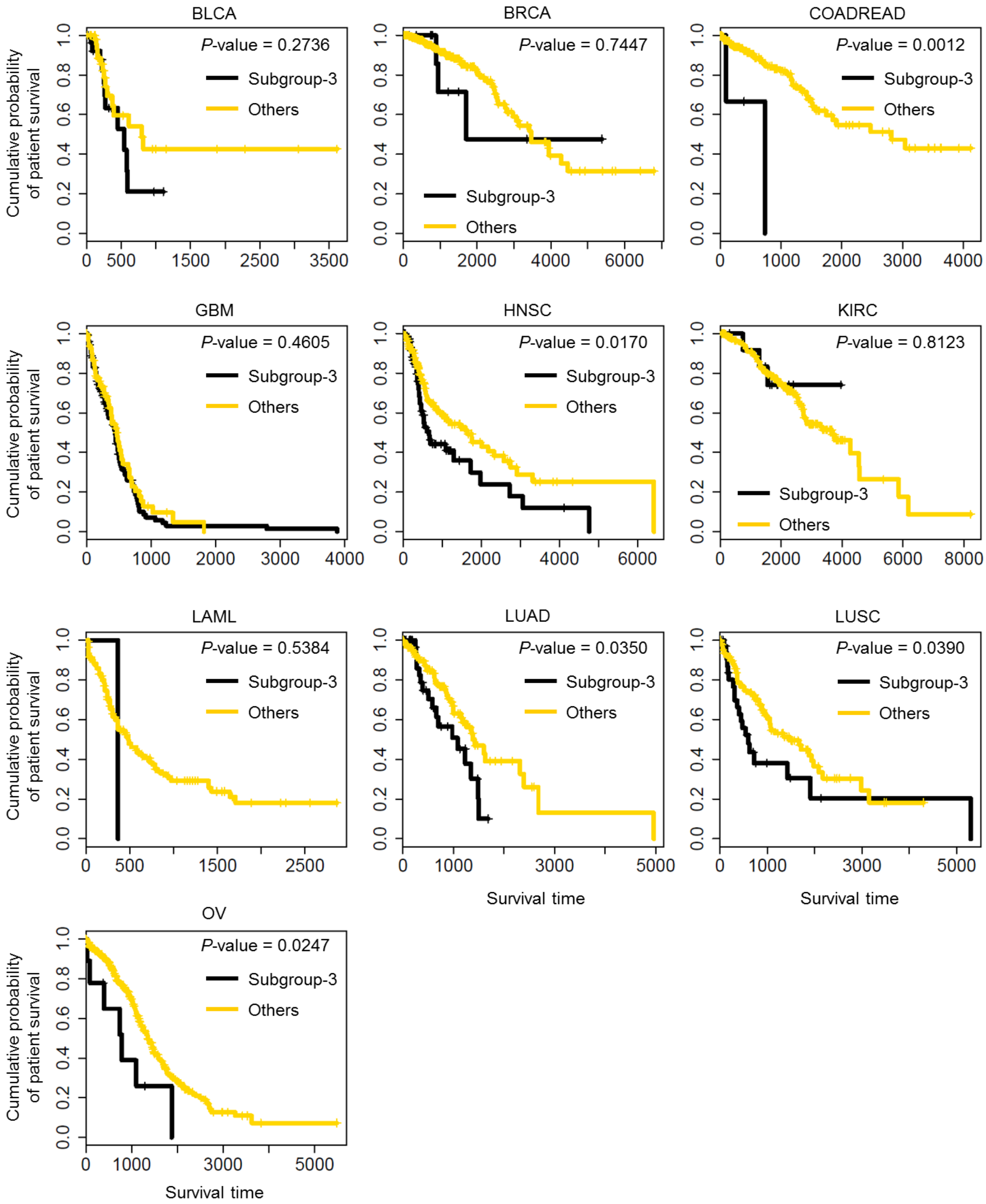

Figure S2. Kaplan-Meier cumulative survival curves of 11 cancer types on the basis of the results of classifying samples into 3 classes. For each cancer type, survival time of patients were compared between those in subgroup-3 and others. *P*-values were derived from the log-rank test. Note that no UCEC patient was classified into subgroup-3 (see Figure 2).

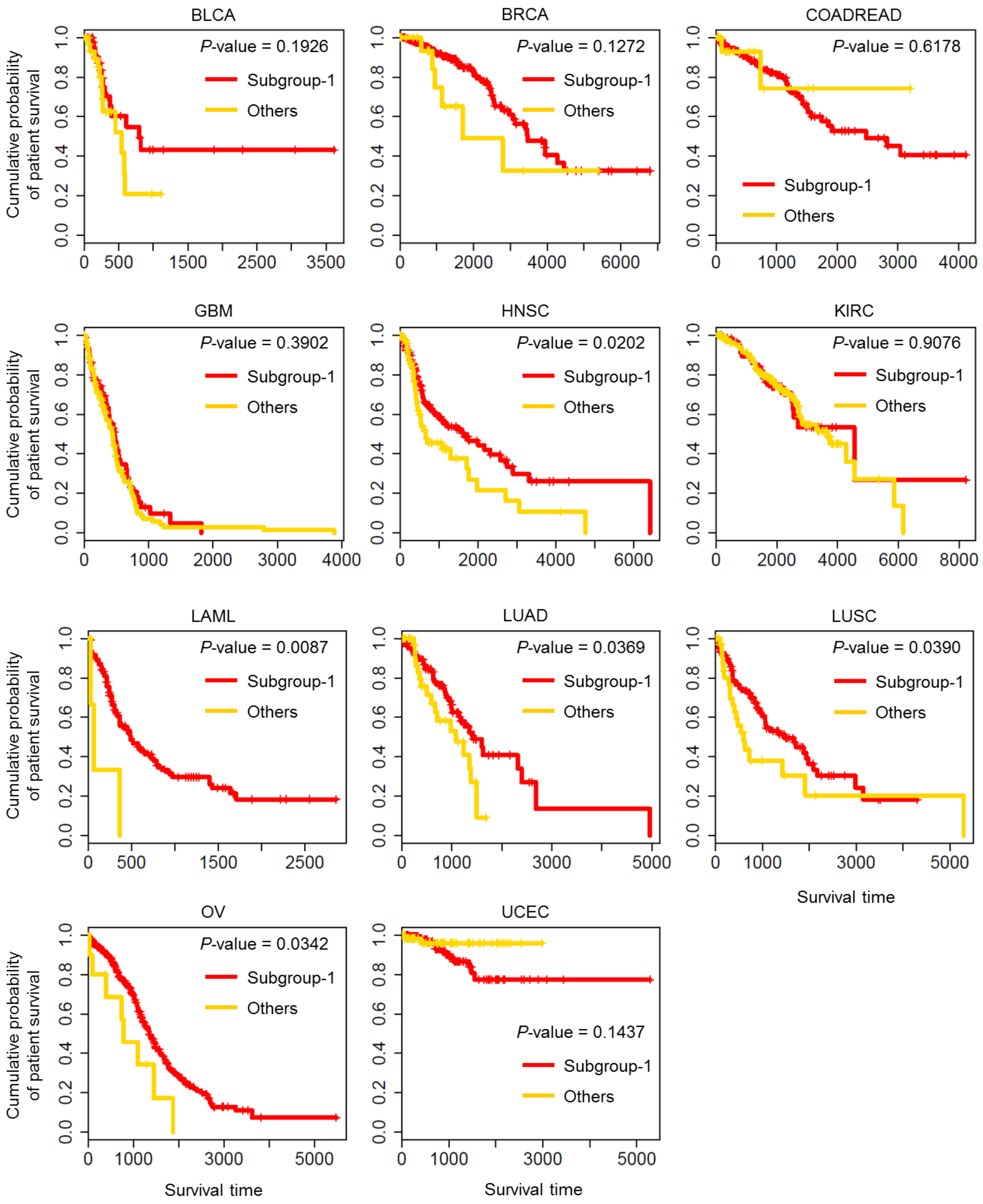

Figure S3. Kaplan-Meier cumulative survival curves of 12 cancer types on the basis of the results of classifying samples into 3 classes. For each cancer type, survival time of patients were compared between those samples in subgroup-1 and others. *P*-values were derived from the log-rank test.

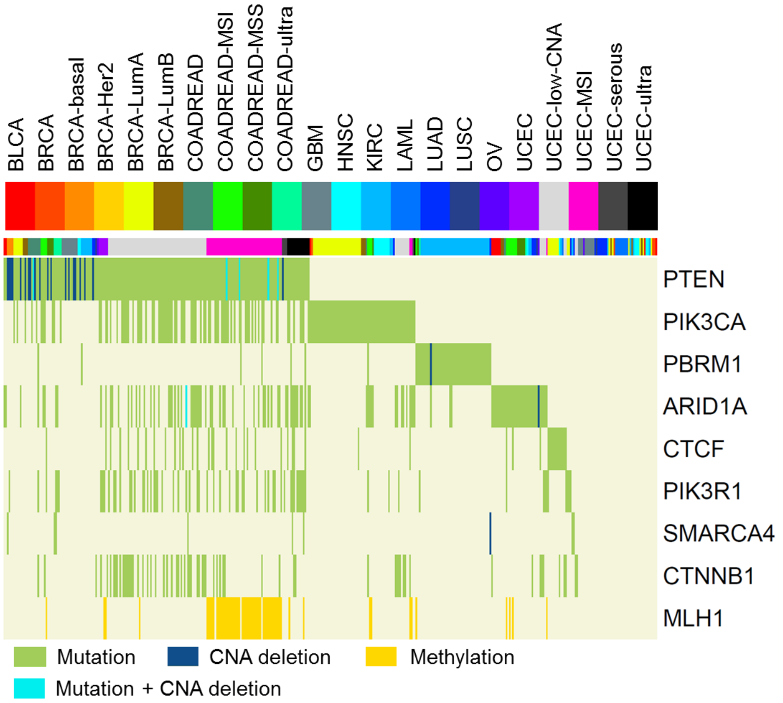

Figure S4. Landscape of genomic alteration patterns of patients in subgroup-1. Each row denotes a gene and each column indicates a sample. The cancer types of patients are denoted by different color at the top of the plot. Subtypes of three cancers are used. BRCA-Her2 stands for the Her2+ breast cancer subtype, while BRCA stands for the breast cancer samples with no subtype information. Different kinds of genetic alterations are denoted by different color at the bottom. Similar settings have been used for Figure S5 to S12 for subgroup-2 to 9.

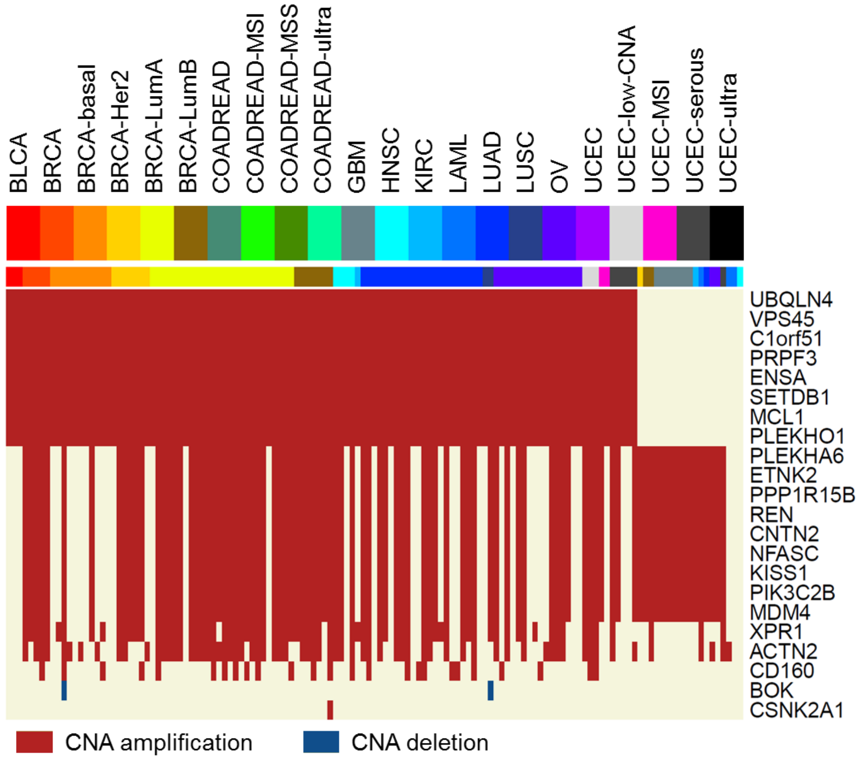

Figure S5.

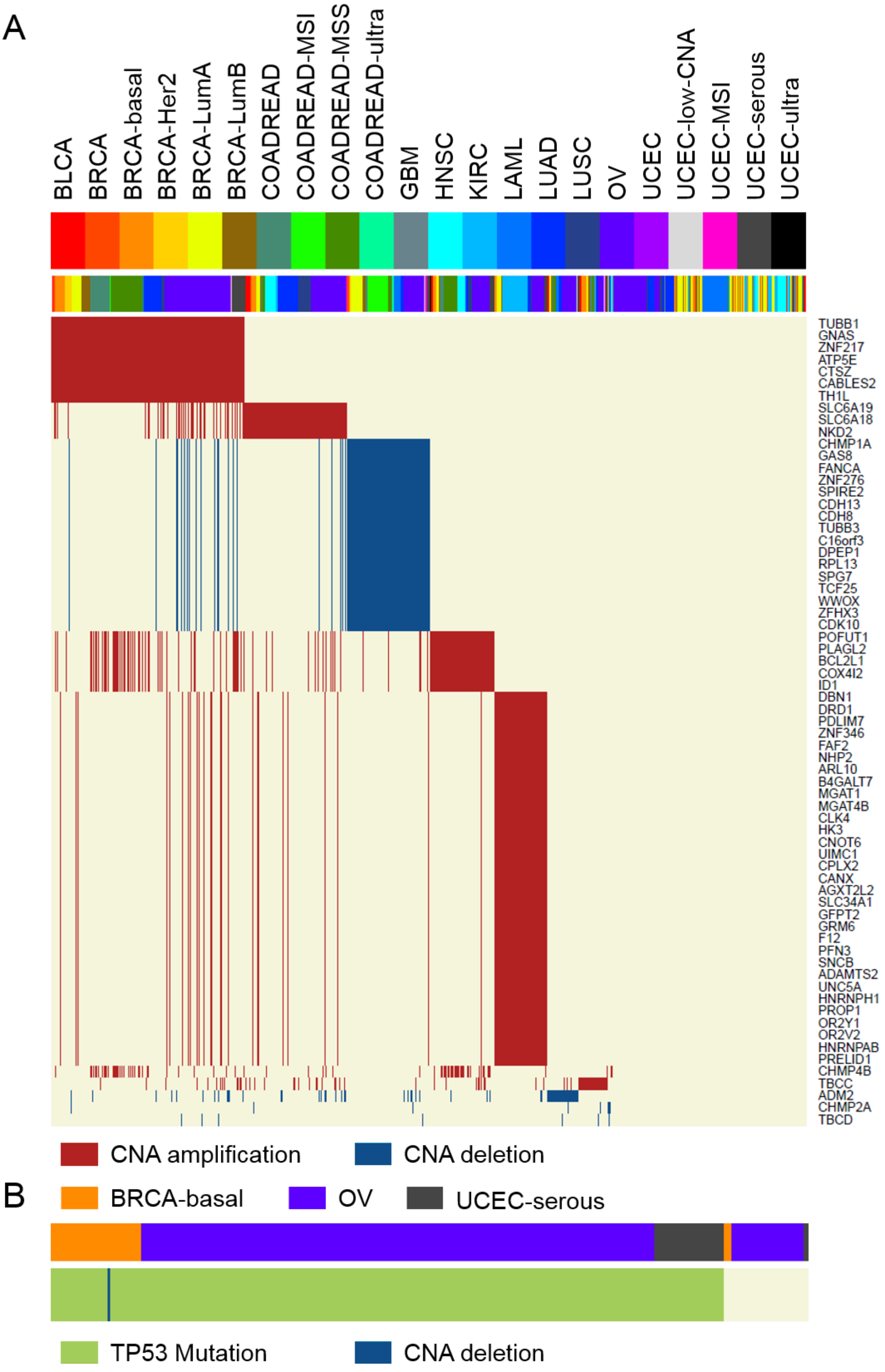

Figure S6. (A) Similar with Figure S4 for subgroup-3. (B) The three enriched cancer types (BRCA-basal, UCEC-serous and OV) in subgroup-3 shared with a high mutation rate of TP53 (88.4%).

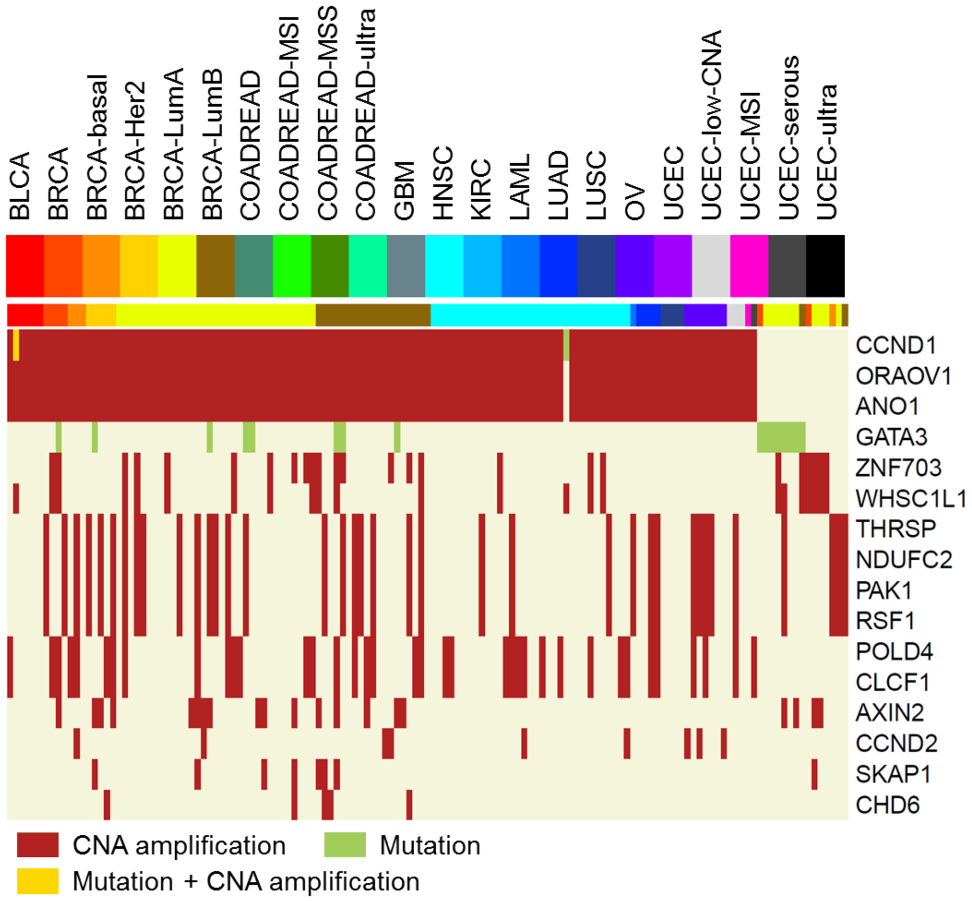

Figure S7.

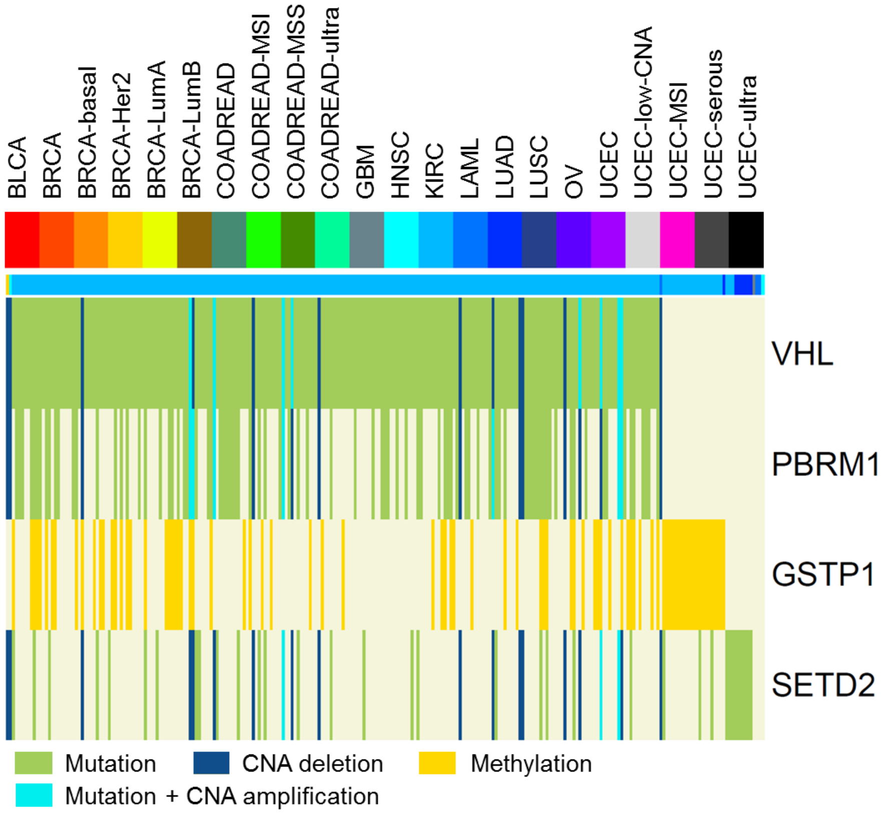

Figure S8.

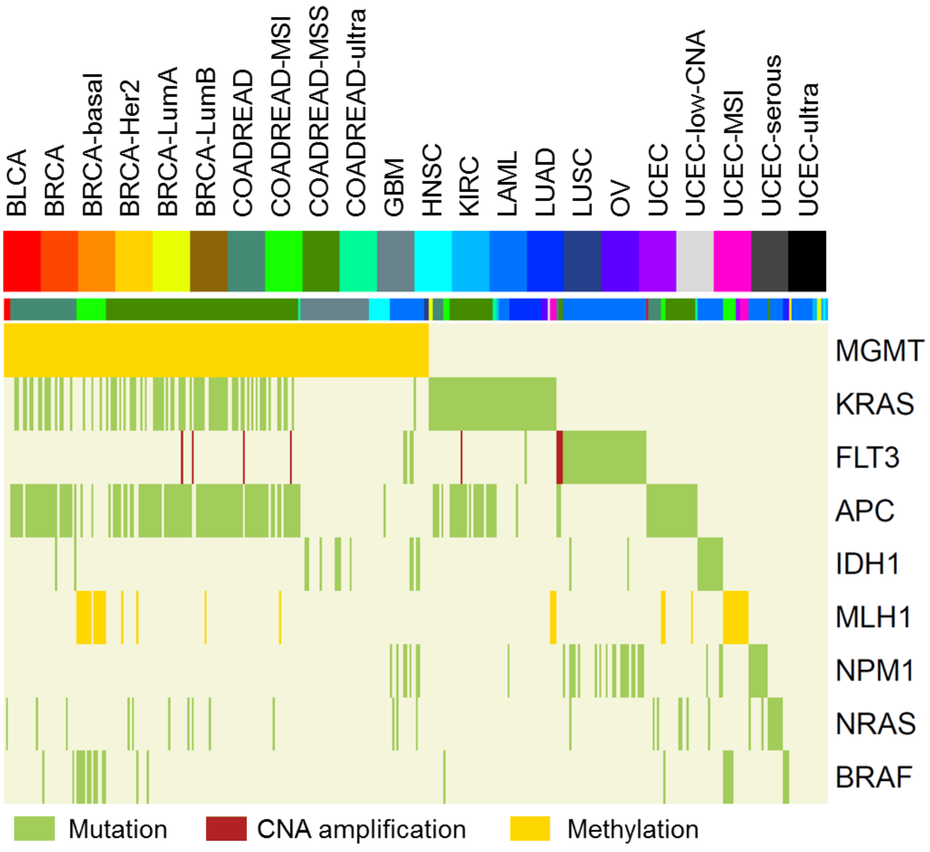

Figure S9.

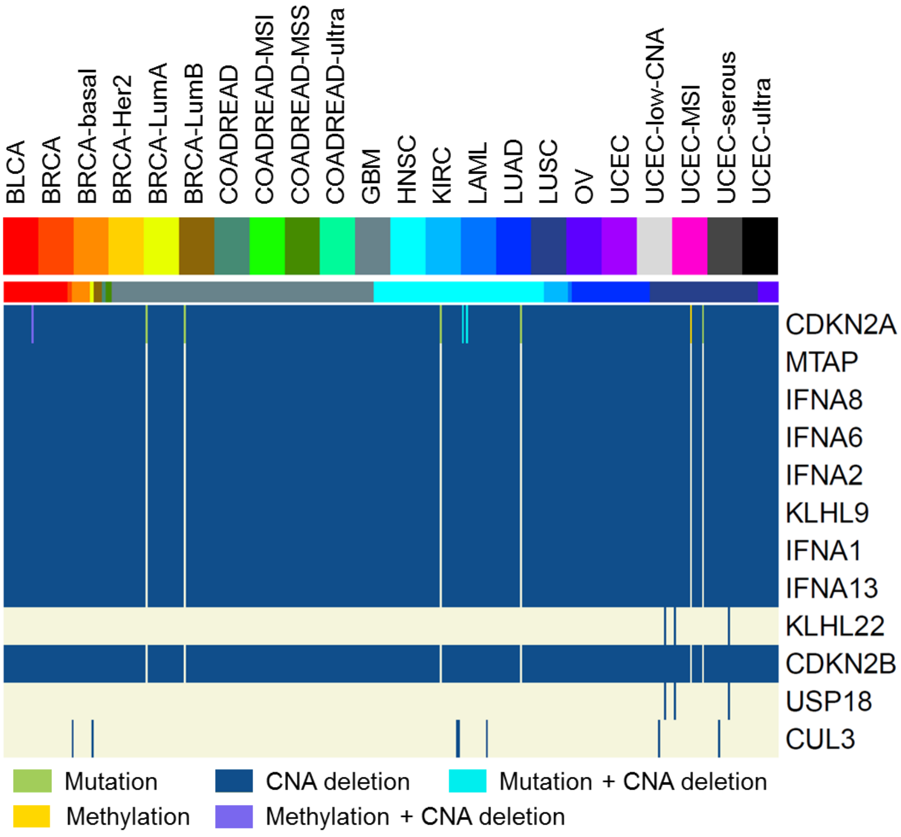

Figure S10.

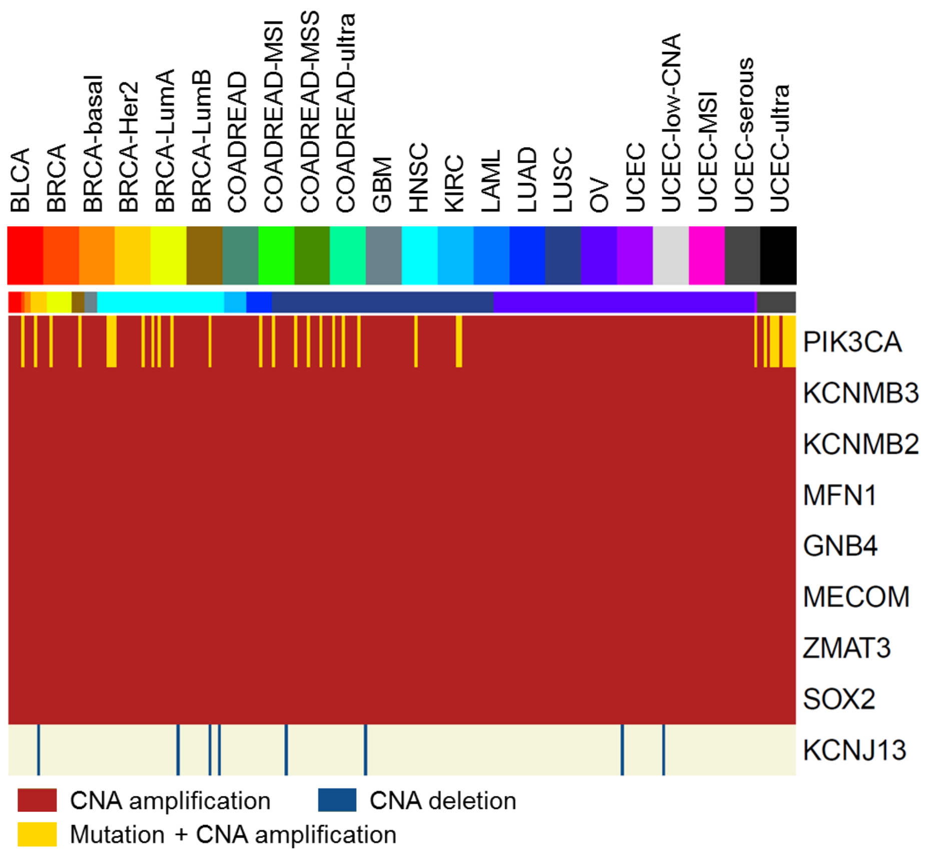

Figure S11.

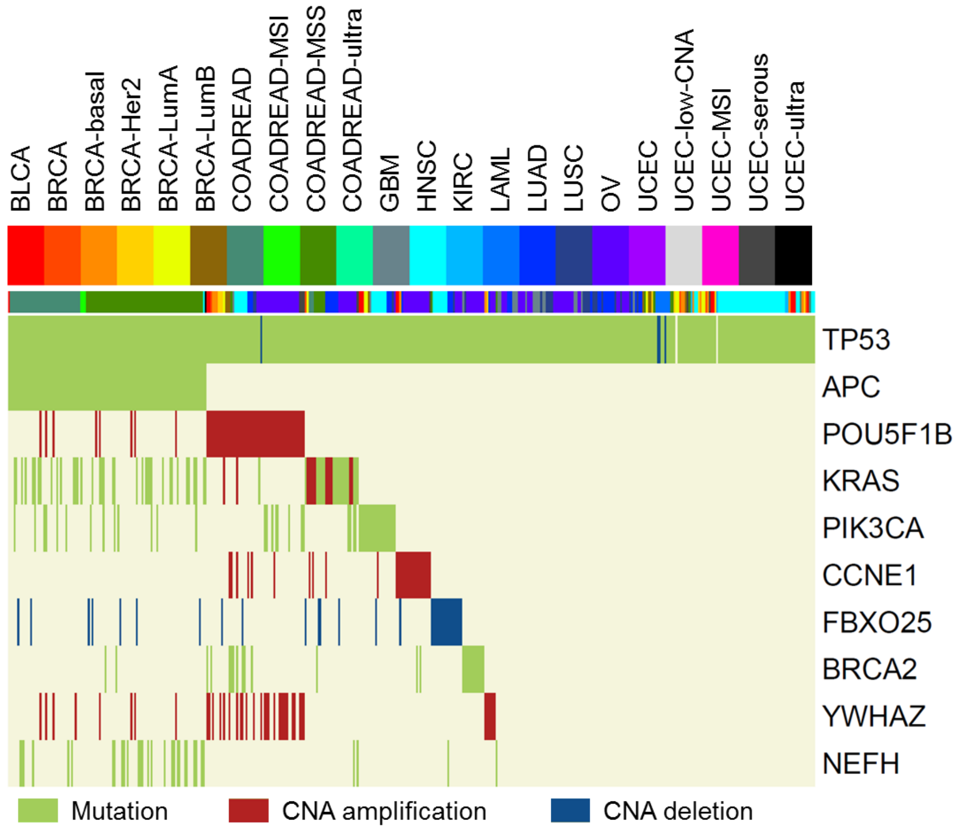

Figure S12.

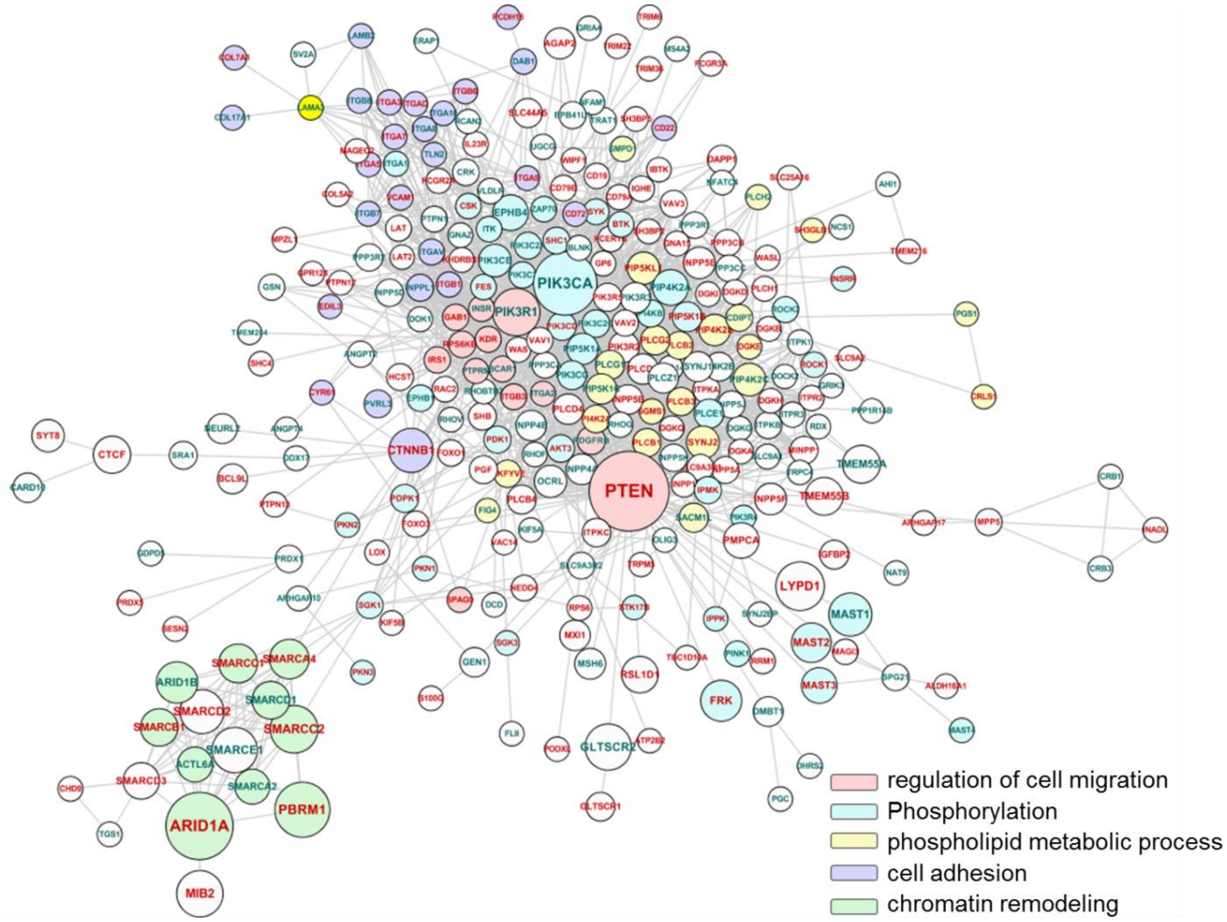

Figure S13. Network modules consisting of significant differential altered genes of subgroup-1. The node size denotes the network propagation score, and the node color corresponds to different biological functional terms. The node labels of genes with significant mRNA expression changes (q-value < 0.05) in subgroup-1 comparing to other patients were marked with green. Similar settings have been used in Figure S14-S21 for subgroup-2 to 9.

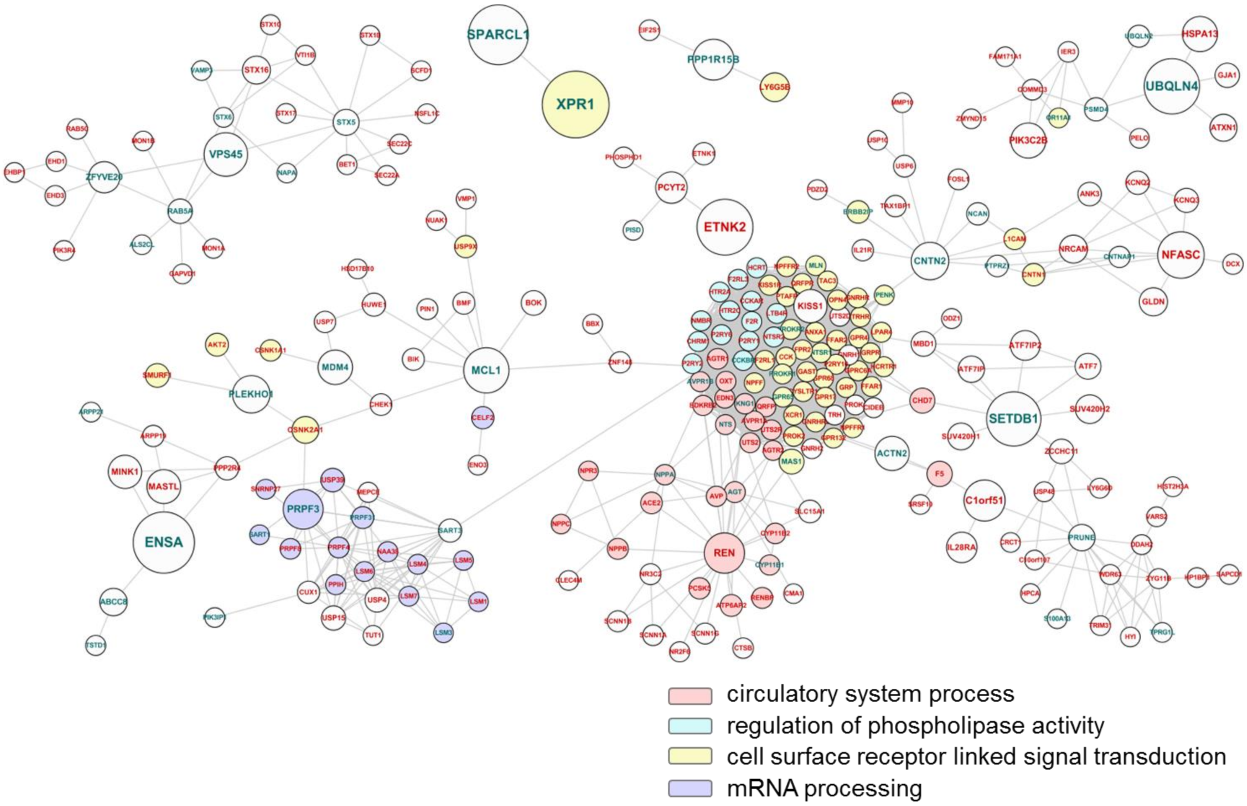

Figure S14.

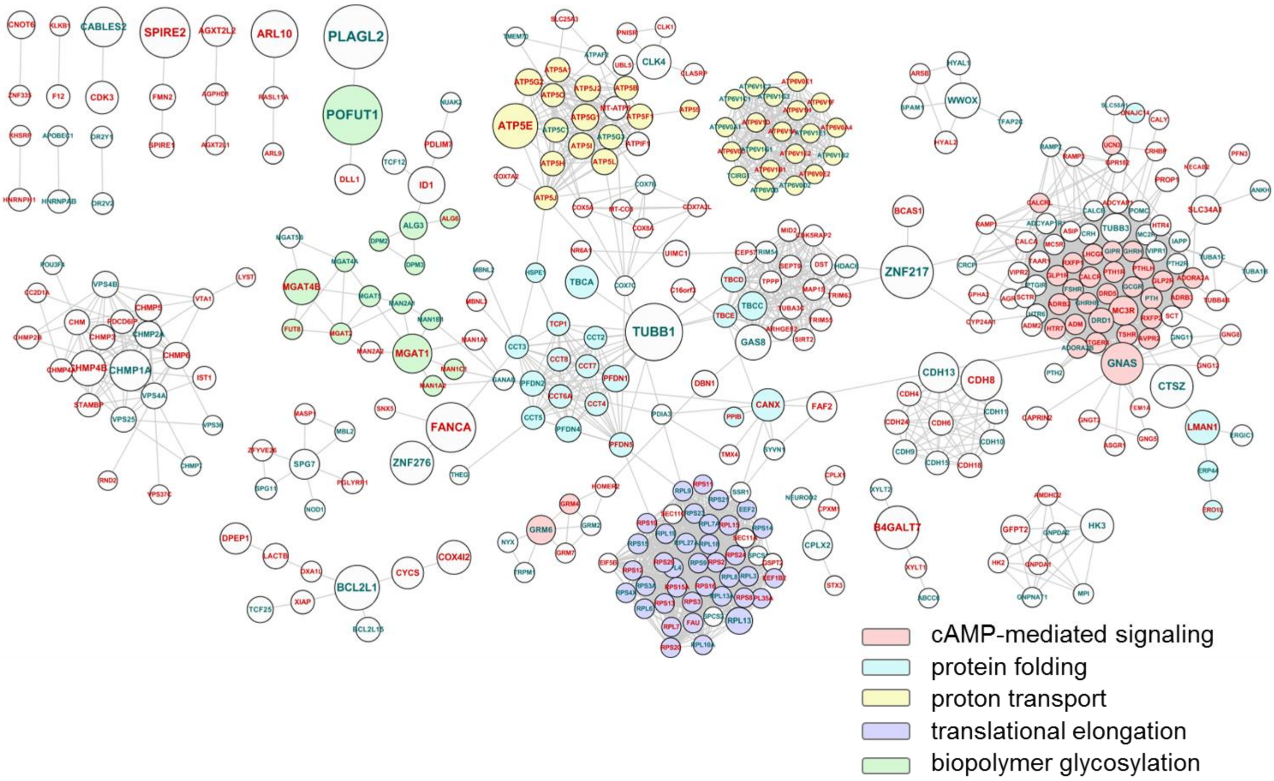

Figure S15.

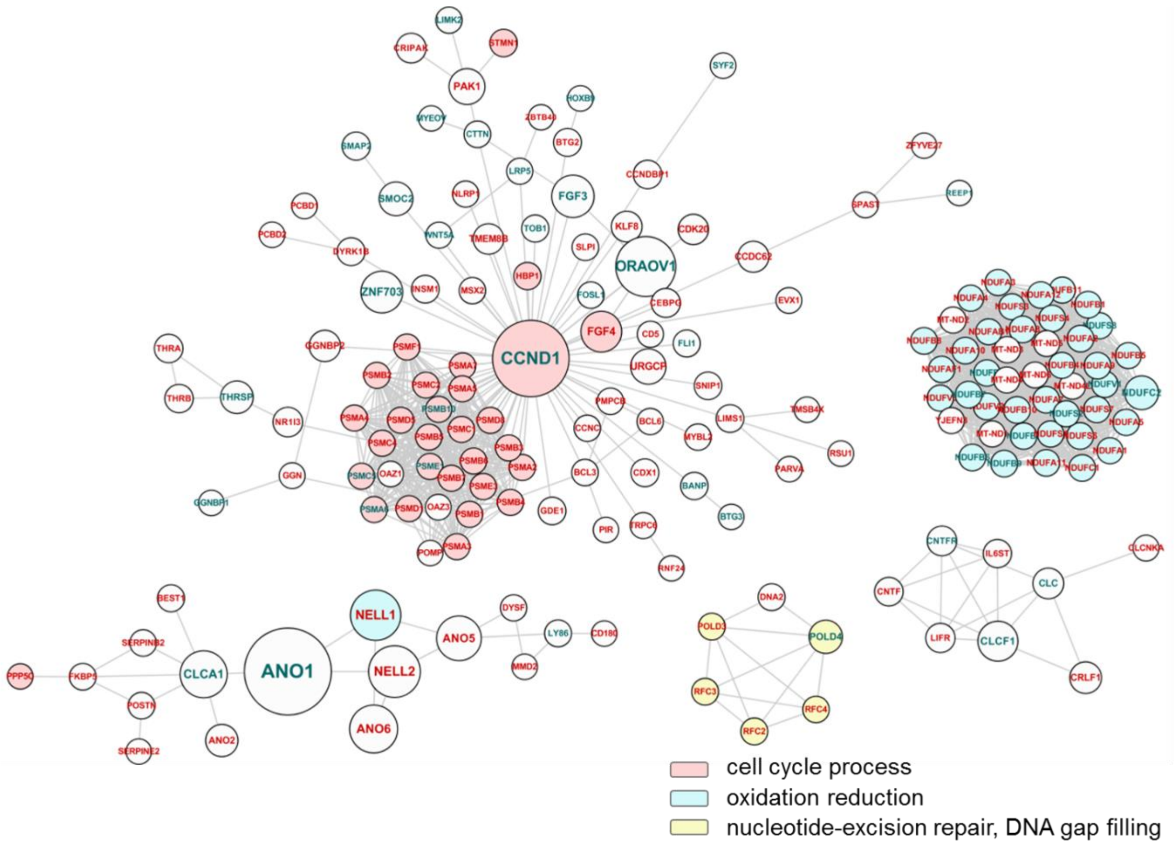

Figure S16.

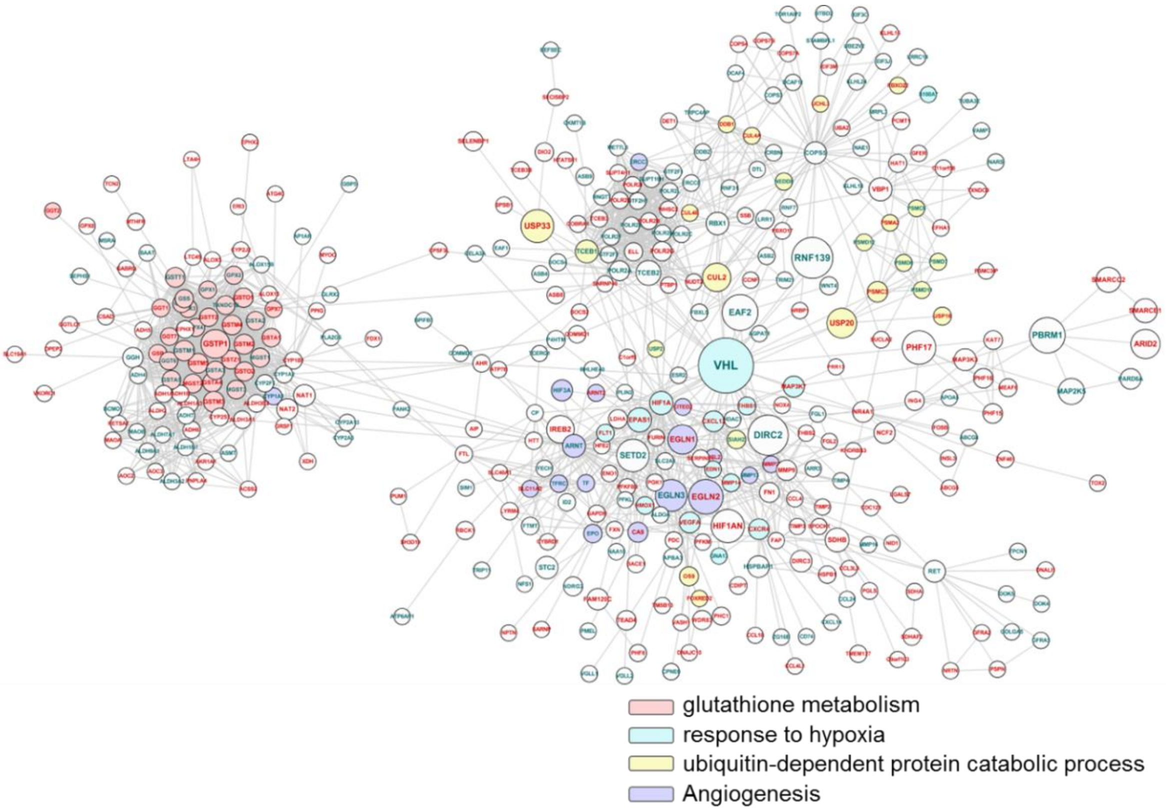

Figure S17.

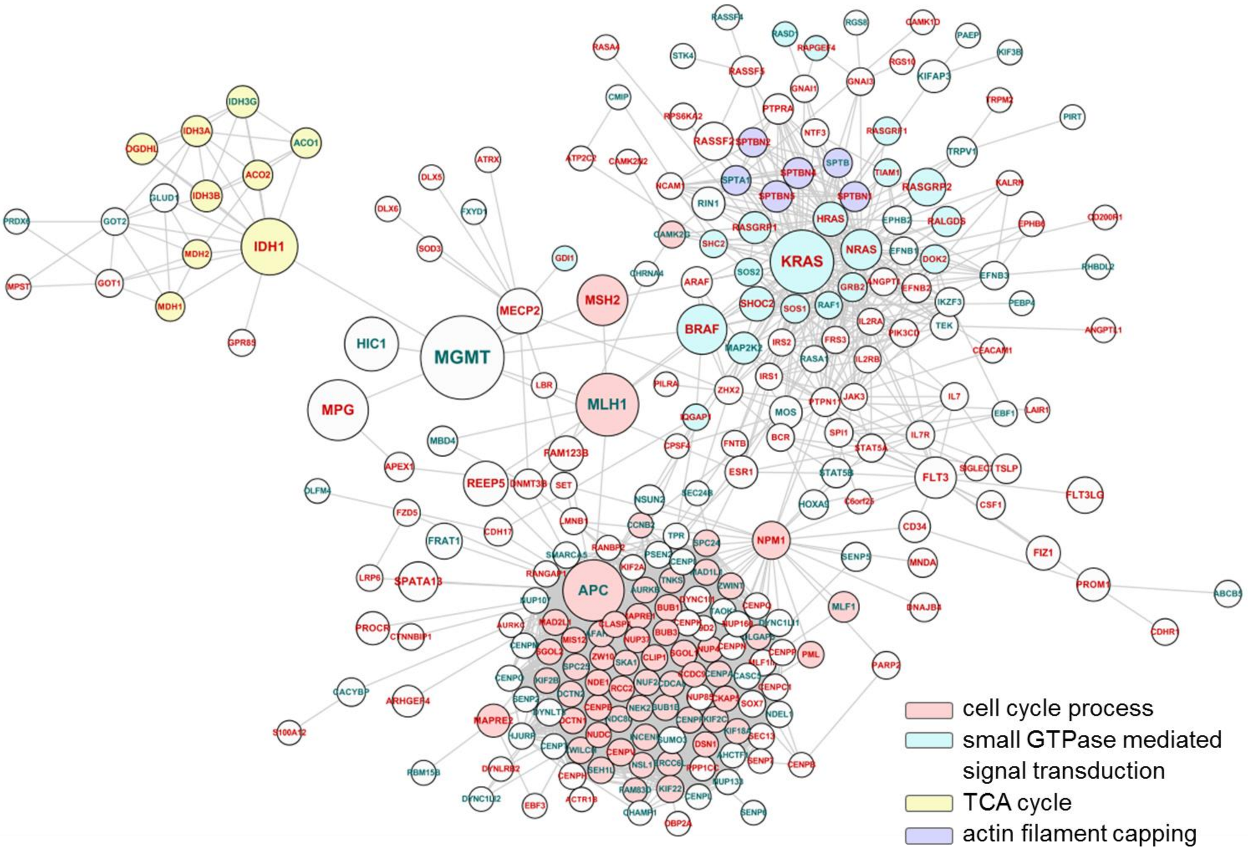

Figure S18.

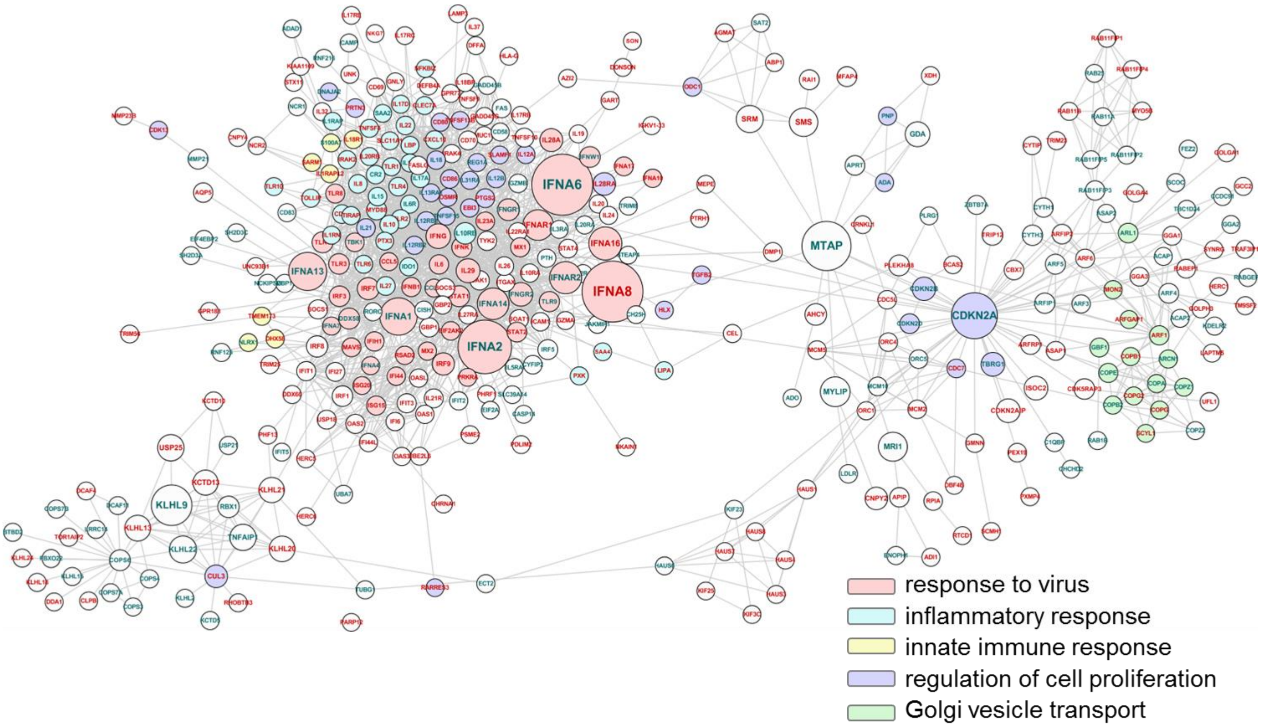

Figure S19.

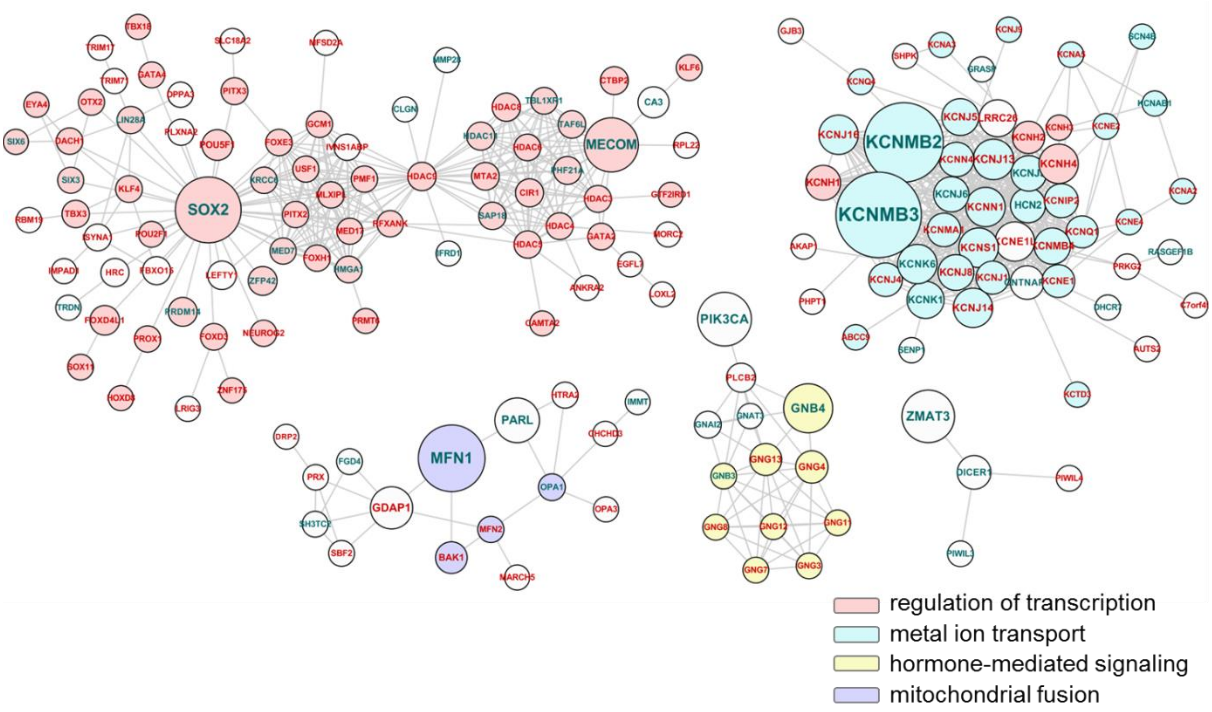

Figure S20.

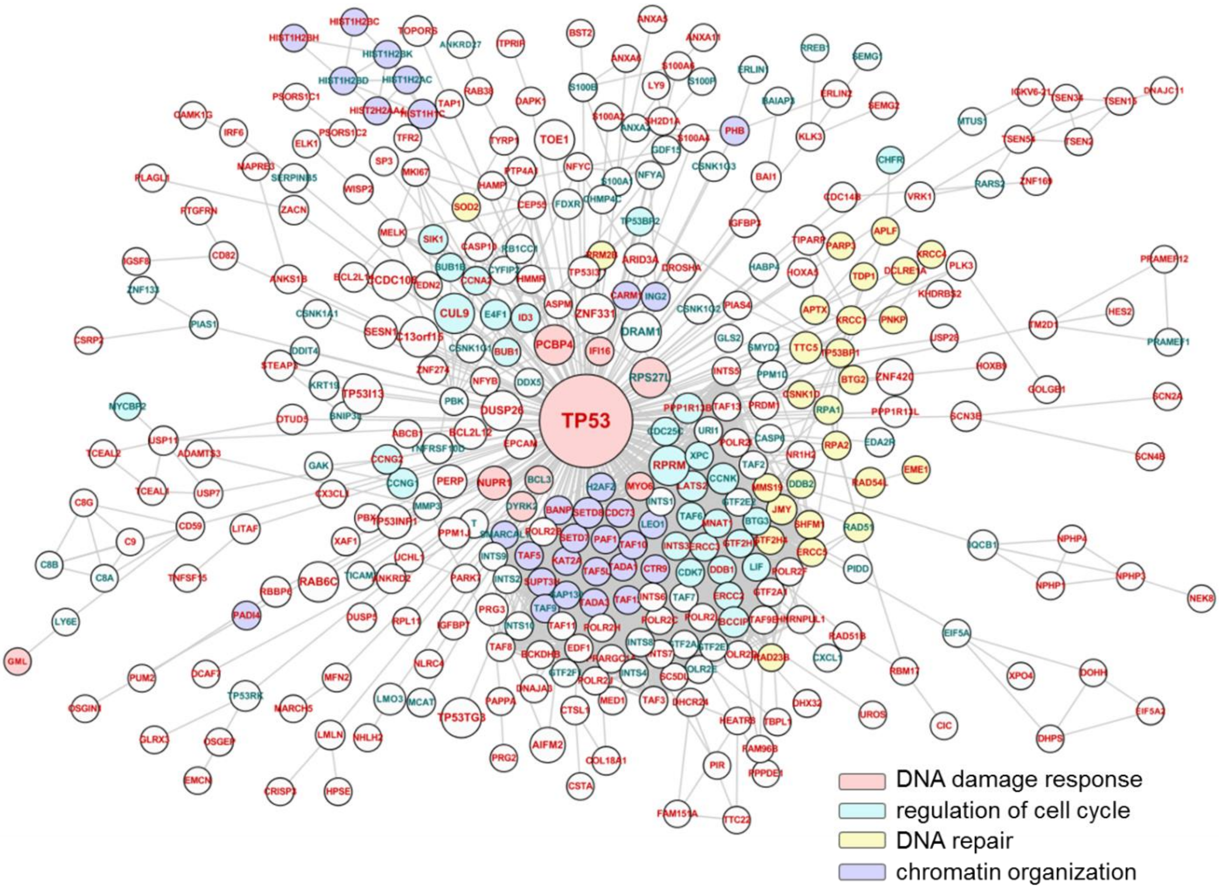

Figure S21.

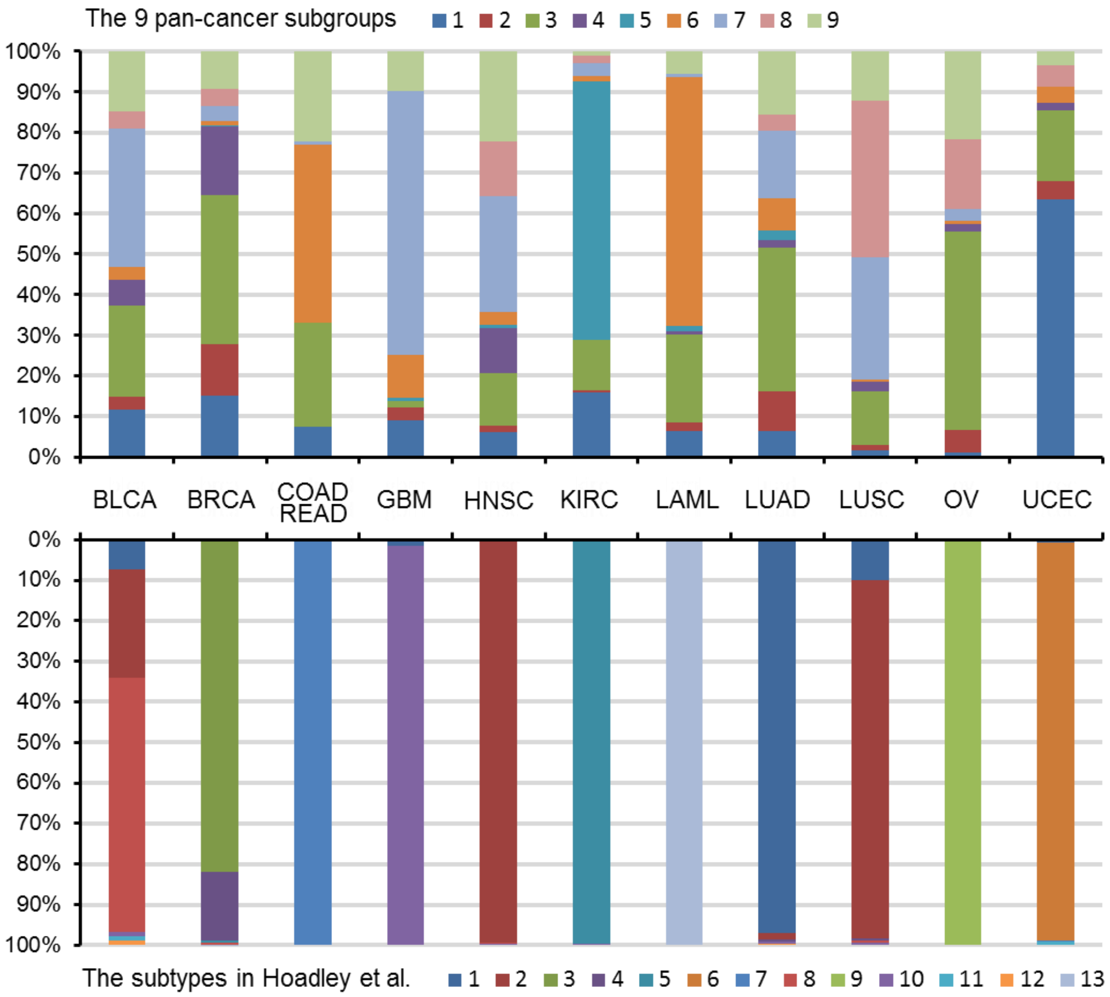

Figure S22. The distributions of the 12 cancer types (COAD and READ were treated as one type) under our pan-cancer classification and that of Hoadley *et al*. Above: our pan-cancer classification (PC9). Below: the major pan-cancer classification by Hoadley *et al.* 2631 samples were involved in both classifications for this comparative analysis.

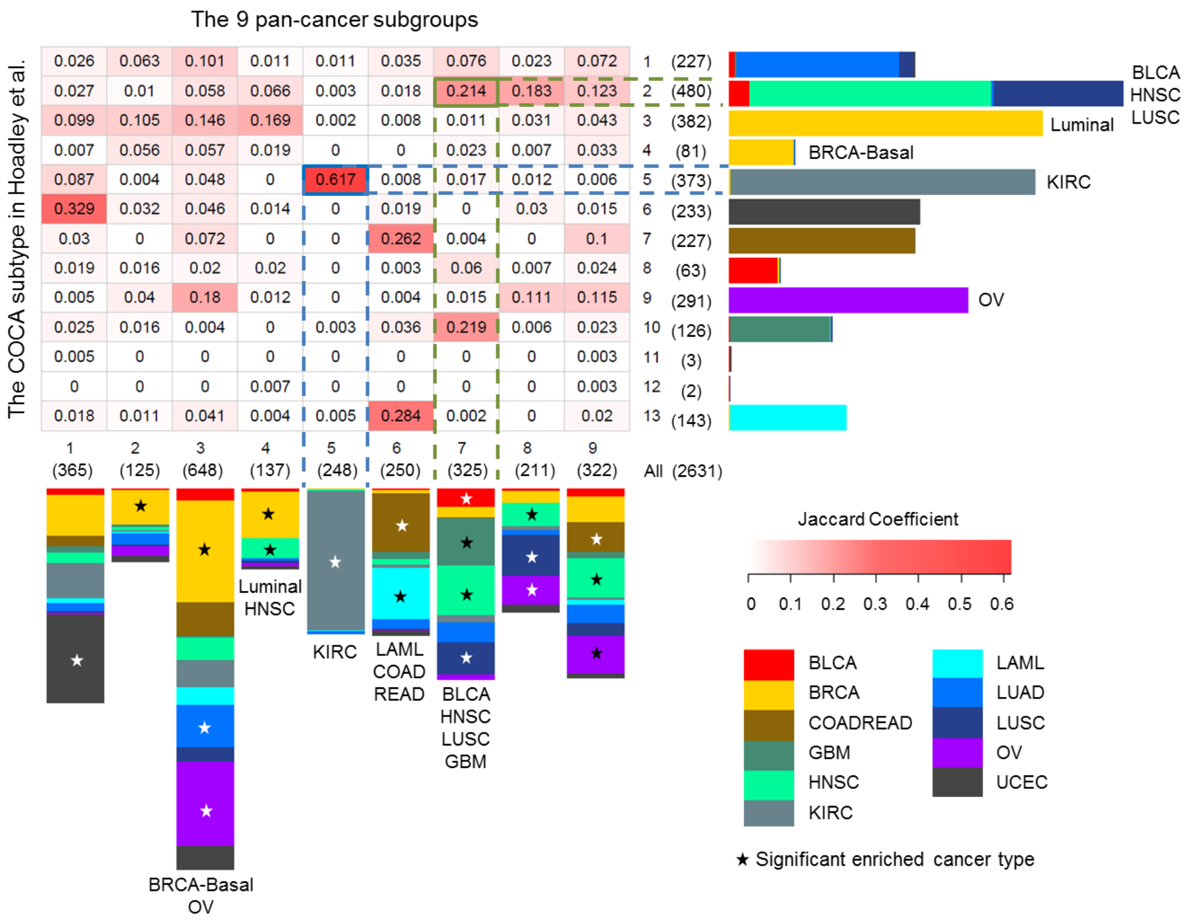

Figure S23. Comparison of our pan-cancer classification and that of Hoadley *et al* on the 2631 samples involved in both studies. The rows of the heatmap denote the 13 classes identified in Hoadley *et al*, and the columns denote the 9 pan-cancer subgroups in our study. The pairwise overlaps of patients in the two classifications are measured by Jaccard coefficient. For each subgroup of the two classifications, a cumulative bar plot is used to indicate the composition of the cancer types with different color. The number in the bracket and the length of the bar plot denote the sample size of each cohort. For subgroups in our classifications, significant enriched cancer types are marked with star (Pearson's chi-squared test, FDR < 0.05).

We evaluated the robustness of our classification of the 9 essential pan-cancer subgroups. To this end, we performed random subsamplings of the samples and reclassified the reduced dataset into 9 categories with the same calculation procedure. The subsamplings were conducted by randomly sampling 95%, 90%, 85% and 80% of the samples while keeping the original proportion of each cancer type. The classification results of the subsampling sets were compared to the original one using the Jaccard coefficient. For example, in order to compare the consistency of the classifications of the whole dataset and 80% subsampling, we extracted the classification results of the same 80% samples on the whole dataset for comparisons and each subgroup calculated on the reduced dataset were paired with a subgroup calculated on the whole dataset which get the maximal Jaccard coefficient. At last, the overall assessment of the robustness of the pan-cancer subgroups was scored with the average of the 9 Jaccard coefficients.

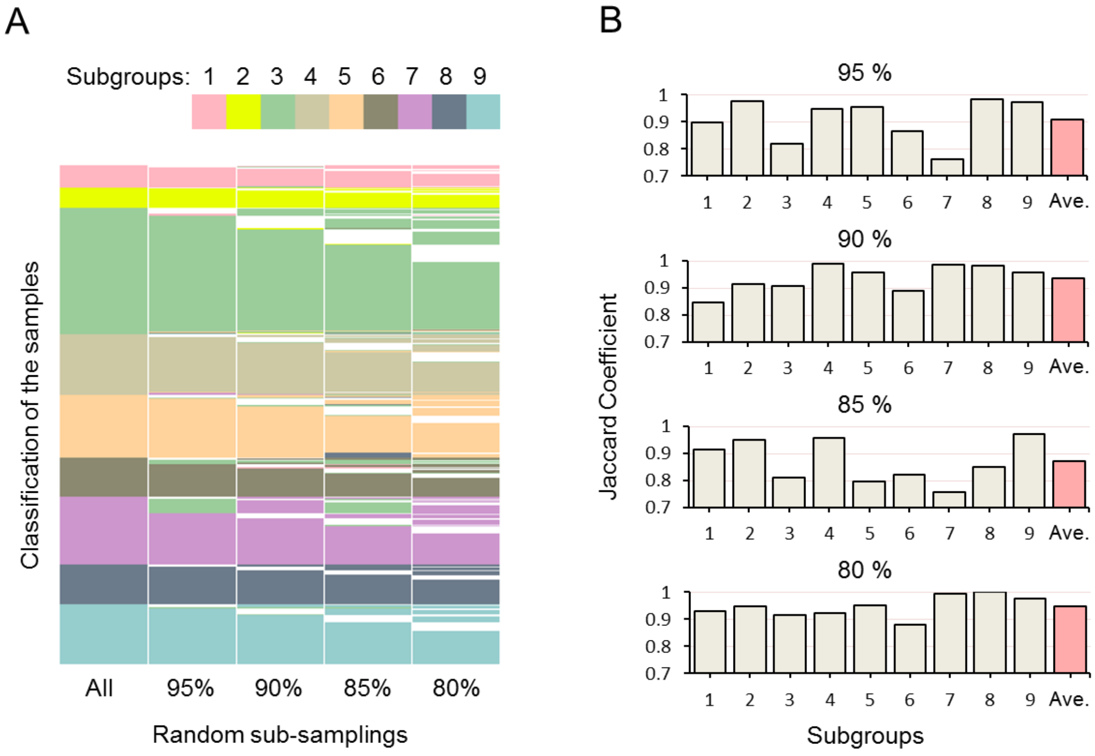

Figure S24. (A) Landscape of the classifications of different random subsamplings. Each row denotes a sample, and each column presents a sub-sampling. Different colors indicate the 9 different subgroups. (B) Comparison of the classification results of different sub-samplings with the original results using the Jaccard coefficient. The Ave. denotes the average of the 9 Jaccard coefficients on each subgroup.
